# Supplementary material for: Protocol of BEYOND trial: Clinical BEnefit of sodium-glucose cotransporter-2 (SGLT-2) inhibitors in rhYthm cONtrol of atrial fibrillation in patients with diabetes mellitus
Source: PLoS One. 2023 Jan 18;18(1):e0280359. doi: 10.1371/journal.pone.0280359 (PMC9847966; doi:10.1371/journal.pone.0280359)
Supplement: S1 File — (DOC) [file pone.0280359.s001.doc]

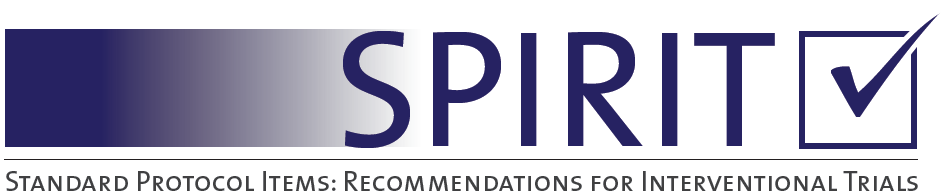


SPIRIT 2013 Checklist: Recommended items to address in a clinical trial protocol and related documents*

| Section/item | ItemNo | Description |
| --- | --- | --- |
| **Administrative information** | | |
| Title | 1 | Descriptive title identifying the study design, population, interventions, and, if applicable, trial acronym   - It is a multicenter study with a prospective, open, blinded endpoint design. It is a 1:1 randomized and controlled study that compares the rhythm control effect of SGLT-2 inhibitor therapy to other oral hypoglycemic agents in patients with AF and DM |
| Trial registration | 2a | Trial identifier and registry name. If not yet registered, name of intended registry   - Clinical BEnefit of Sodium-glucose cotransporter-2 (SGLT-2) inhibitors in rhYthm cONtrol of atrial fibrillation in patients with Diabetes mellitus (BEYOND trial) |
|  | 2b | All items from the World Health Organization Trial Registration Data Set   - ClinicalTrials.gov - NCT05029115 |
| Protocol version | 3 | Date and version identifier   - Version 1.0: 31/08/2021 |
| Funding | 4 | Sources and types of financial, material, and other support   - This trial is supported by Basic Science Research Program through the National Research Foundation of Korea (NRF) funded by the Ministry of Science, ICT & Future Planning (NRF-2017R1E1A1A01078382), and by the Korea Medical Device Development Fund grant funded by the Republic of Korea government (the Ministry of Science and ICT, the Ministry of Trade, Industry and Energy, the Ministry of Health & Welfare, the Ministry of Food and Drug Safety) (Project Number: 9991006899). |
| Roles and responsibilities | 5a | Names, affiliations, and roles of protocol contributors   - Kyuhyun Lee (College of Medicine, Ewha Womans University, Seoul, Republic of Korea) - Soo Kyoung Lee (College of Medicine, Ewha Womans University, Seoul, Republic of Korea) - Juyeon Lee (College of Medicine, Ewha Womans University, Seoul, Republic of Korea) - Bo Kyung Jeon (Department of Cardiology, Ewha Womans University Medical Center, Seoul, Republic of Korea) - Tae-Hoon Kim (Yonsei University Health System, Seoul, Republic of Korea) - Hee Tae Yu (Yonsei University Health System, Seoul, Republic of Korea) - Jung Myung Lee (Kyung Hee University, Medical Center, Seoul, Republic of Korea) - Jin-Kyu Park (Department of Cardiology, Hanyang University Seoul Hospital, Seoul, Republic of Korea) - Yong-Soo Baek (Inha University College of Medicine and Inha University Hospital, Incheon, Republic of Korea) - Dong Hyeok Kim (Department of Cardiology, Ewha Womans University Medical Center, Seoul, Republic of Korea) - Jaemin Shim (Korea University College of Medicine, Anam Hospital, Seoul, Republic of Korea) - Boyoung Joung (Yonsei University Health System, Seoul, Republic of Korea) - Moon-Hyoung Lee (Yonsei University Health System, Seoul, Republic of Korea) - Hui-Nam Pak (Yonsei University Health System, Seoul, Republic of Korea) - Junbeom Park (Department of Cardiology, Ewha Womans University Medical Center, Seoul, Republic of Korea) - K.L, S.K.L and J.L wrote the detailed paper of protocol. J.P initially designed this protocol and B.K.J, T.K, H.T.Y, J.M.L, J.P, Y.B, D.H.K, J.S, B.J, M.L, H.P and J.P edited it to more practical thing. K.L, S.K.L, J.L and J.P had primary responsibility for final content. |
|  | 5b | Name and contact information for the trial sponsor   - Not applicable |
|  | 5c | Role of study sponsor and funders, if any, in study design; collection, management, analysis, and interpretation of data; writing of the report; and the decision to submit the report for publication, including whether they will have ultimate authority over any of these activities   - This funding source had no role in the design of this study and will not have any role during its execution, analyses, interpretation of the data, or decision to submit results. |
|  | 5d | Composition, roles, and responsibilities of the coordinating centre, steering committee, endpoint adjudication committee, data management team, and other individuals or groups overseeing the trial, if applicable (see Item 21a for data monitoring committee)   - Research physician - Preparation of protocol - Recruitment participant and getting informed consent - IRB of each involved Hospital - Check interim report annually and ethical consistency - Adverse Event-review committee - Judged adverse event every twice a year |
| Introduction |  |  |
| Background and rationale | 6a | Description of research question and justification for undertaking the trial, including summary of relevant studies (published and unpublished) examining benefits and harms for each intervention   - SGLT-2 inhibitor, traditionally used for glycemic control, has several beneficial effects that can help manage heart failure (HF). SGLT-2 inhibitors reduce the risk of cardiovascular mortality in patients with HF. As atrial fibrillation (AF) is closely associated with HF and diabetes mellitus (DM) is a risk factor for AF, we assume that SGLT-2 inhibitors will also show therapeutic benefits regarding AF, especially for rhythm control. |
|  | 6b | Explanation for choice of comparators   - Control group will use hypoglycemic agents except SGLT-2 inhibitor (e.g., metformin, meglitinides, sulfonylureas, DPP-4 inhibitors, GLP-1 receptor agonists, α-glucosidase inhibitors, and thiazolidinediones) to control their serum glucose. |
| Objectives | 7 | Specific objectives or hypotheses   - The primary hypothesis of this study is that the SGLT-2 inhibitor is superior to other oral hypoglycemic medications for rhythm control in patients with AF and DM, diagnosed within the prior year. |
| Trial design | 8 | Description of trial design including type of trial (eg, parallel group, crossover, factorial, single group), allocation ratio, and framework (eg, superiority, equivalence, noninferiority, exploratory)   - Parallel group, 1:1, exploratory trial |
| Methods: Participants, interventions, and outcomes | | |
| Study setting | 9 | Description of study settings (eg, community clinic, academic hospital) and list of countries where data will be collected. Reference to where list of study sites can be obtained   - Multicenter study consisted of several academic hospital in South Korea - Ewha Womans University Mokdong Hospital - Yonsei University Sinchon Severance Hospital - Korea University Anam Hospital - Hanyang University Hospital - Kyung Hee University Hospital - Inha University Hospital - Ewha Womans University Seoul Hospital - Yonsei University Yongin Severance Hospital |
| Eligibility criteria | 10 | Inclusion and exclusion criteria for participants. If applicable, eligibility criteria for study centres and individuals who will perform the interventions (eg, surgeons, psychotherapists)   - Inclusion Criteria  1. At least one episode of AF that is documented during the prior year by any kind of ECG recording. 2. Type 2 DM was diagnosed (HbA1c > 6.5%) and the patient was using oral hypoglycemic agents only for glycemic control. 3. At least 18 years old, but not older than 80 years. 4. Normal ECG parameters, measured in sinus rhythm (QRS width ≤ 120 ms, QTc interval < 440 ms, and PQ interval ≤ 210 ms in a 12-lead ECG). 5. Be able and willing to give informed consent.  - Exclusion Criteria  1. Any disease that limits life expectancy to under 1 year 2. Subject for another clinical trial within the past 2 months 3. Under 18 years old or over 80 years 4. Pregnant women 5. Lactating women 6. Drug abuser 7. Type 2 DM treated by recombinant insulin 8. Diagnosis of Type 1 DM, MODY, or secondary DM 9. HbA1c ≥ 12% or HbA1c < 6.5% at diagnosis 10. Previous treatment with any SGLT-2 inhibitor 11. Renal dysfunction (eGFR-CKD-EPI < 30 mL/min/1.73 m^2) 12. Chronic cystitis and/or recurrent genitourinary tract infections (3 or more in the last year) 13. Unexplained hematuria at baseline study 14. Systolic BP > 180 mmHg or diastolic BP > 100 mmHg at baseline study 15. Systolic BP < 95 mmHg at baseline study 16. Previous treatment with AF ablation 17. Acute cardiovascular event [e.g., stroke, acute coronary syndrome (ACS), revascularization, decompensated HF, sustained ventricular tachycardia, return of spontaneous circulation (ROSC)] <8 weeks prior to baseline study 18. Severe valvular disease or have prosthetic valve 19. Treatment with chronic oral steroid (>30 consecutive days) at a dose equivalent to oral prednisolone ≥ 10 mg/d, within the past 1 month 20. History of any malignancy within 5 years 21. Clinically profound hepatic dysfunction 22. Clinically uncontrolled thyroid dysfunction 23. Patients incapable of completing the trial because of any severe medical condition by clinical decision 24. Patients with poor compliance (defined as 80–120%), except for reasonable situations judged by physician |
| Interventions | 11a | Interventions for each group with sufficient detail to allow replication, including how and when they will be administered   - DM control: The subjects will receive oral hypoglycemic agents for glucose control; the type of agents will be determined by randomization. Patients using SGLT-2 inhibitors to control their serum glucose will be specified as the case group, while those using other hypoglycemic agents (e.g., metformin, meglitinides, sulfonylureas, DPP-4 inhibitors, GLP-1 receptor agonists, α-glucosidase inhibitors, and thiazolidinediones) to control their serum glucose will be classified as the control group. The number, type, and dosage of the agents will be individualized at the discretion of the physician in accordance with the 2021 ADA guidelines, considering factors such as the kidney function, age, and cardiovascular status. - AF rhythm control: The rhythm control strategy for AF will be followed, independent of the type of hypoglycemic agents received. This strategy includes two sessions. In session 1, an AAD will be assigned to every participant and taken for at least 3 months. If patients have persistent AF, cardioversion will be performed with AAD. During the follow-up, those with recurrent AF should step up to session 2, which involves treatment with radiofrequency ablation (RFA) or CRYO-balloon ablation (4 pulmonary vein isolation; 4PVI). For participants of session 2, the follow-up will begin with the moment RFA or cryotherapy is applied, which will be regarded as 0-month; follow-up will be conducted every 3 months for the next 12 months. The rhythm control strategy will be conducted as per the clinical guidelines of 2020 ESC and 2019 AHA/ACC/HRS guidelines for AF. |
|  | 11b | Criteria for discontinuing or modifying allocated interventions for a given trial participant (eg, drug dose change in response to harms, participant request, or improving/worsening disease)   - All unexpected medical events or laboratory findings will be checked at every 3-months follow-up, and that adverse events will be assessed and judged twice a year by an independent committee. More than half of the votes should be matched to finalize the decision. |
|  | 11c | Strategies to improve adherence to intervention protocols, and any procedures for monitoring adherence (eg, drug tablet return, laboratory tests)   - Every 3-month follow-up, physician will check-up patient’s compliance with other laboratory profile. |
|  | 11d | Relevant concomitant care and interventions that are permitted or prohibited during the trial   - Any unexpected medical events or laboratory findings are also defined as adverse events. Atrial arrhythmia alone is not classified as an adverse event. Instead, it is considered as the primary and/or secondary outcome. |
| Outcomes | 12 | Primary, secondary, and other outcomes, including the specific measurement variable (eg, systolic blood pressure), analysis metric (eg, change from baseline, final value, time to event), method of aggregation (eg, median, proportion), and time point for each outcome. Explanation of the clinical relevance of chosen efficacy and harm outcomes is strongly recommended   - Primary Outcome: The recurrence rate of AF after performing stepwise rhythm control therapies including anti-arrhythmic drugs (AAD) and ablation - Secondary Outcome  1. Rhythm control   AF-free survival  AF burden at  - 3-month F/U visit, 12-month F/U visit in those who did not step up to session 2  - Immediately before trying ablation, 12-month F/U visit in those who stepped up to session 2  Percentage of patients undergoing ablation within a year  Sinus rhythm maintenance   1. Diameter of LA 2. NT-proBNP 3. Symptom score (EHRA score) 4. Quality of life (EQ-5D, SF-12) |
| Participant timeline | 13 | 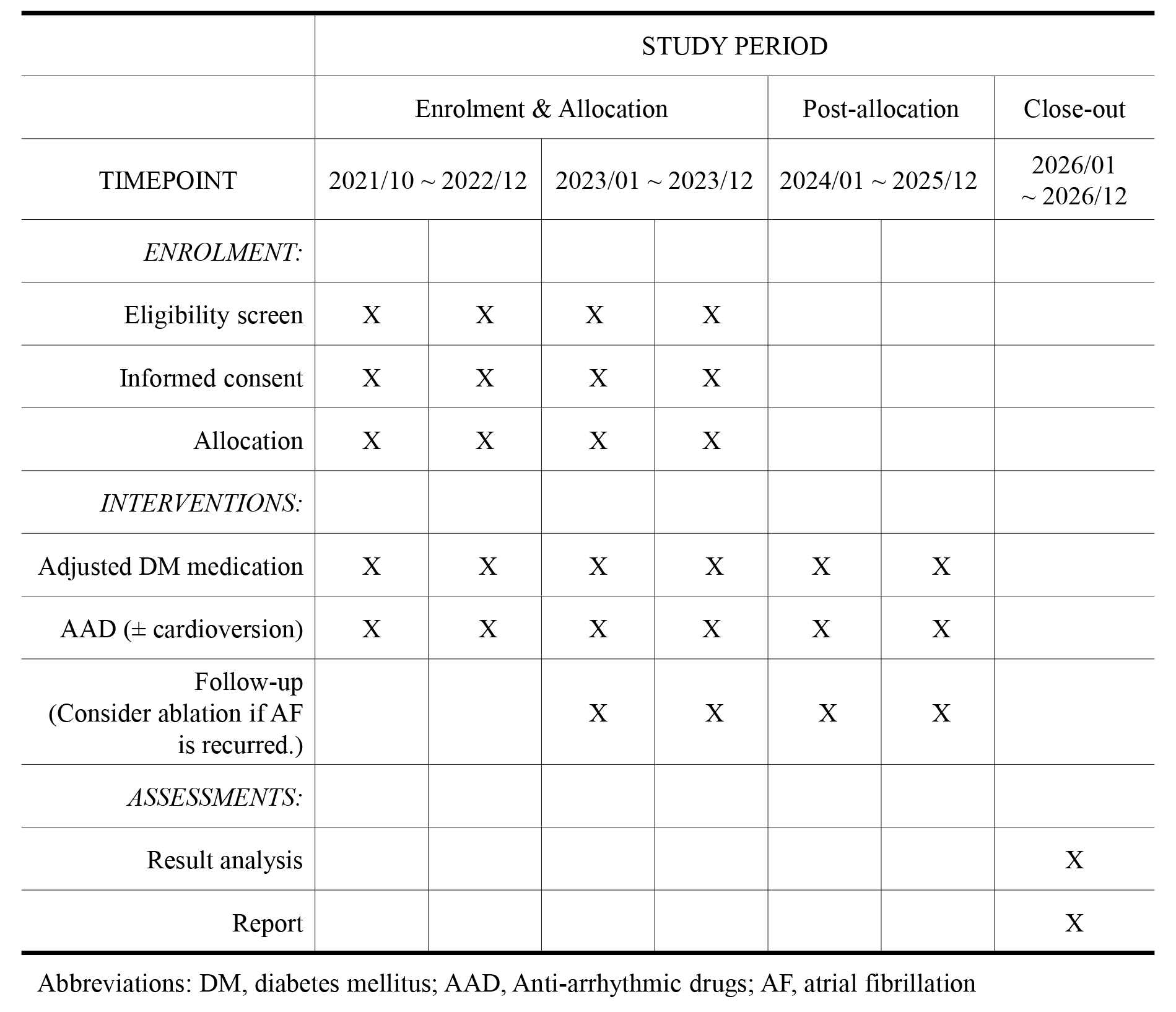Time schedule of enrolment, interventions (including any run-ins and washouts), assessments, and visits for participants. A schematic diagram is highly recommended (see Figure) |
| Sample size | 14 | Estimated number of participants needed to achieve study objectives and how it was determined, including clinical and statistical assumptions supporting any sample size calculations   - In this study, 716 patients will be enrolled from 7 tertiary medical centers. The sample size was determined based on the primary outcome, i.e., the SGLT-2 inhibitors should show 40% reduction in AF after a year of treatment and 50% of AAD users should step up to the ablation therapy. Assuming 10% follow-up loss, the sample size of 716 patients (358 in each group) is expected to achieve 80% power and an alpha-level of 5% to detect the difference. |
| Recruitment | 15 | Strategies for achieving adequate participant enrolment to reach target sample size   - Patient will be enrolled at outpatient of cardiology and endocrinology department of multiple medical center in South Korea until adequate sample size is achieved. |
| **Methods: Assignment of interventions (for controlled trials)** | | |
| Allocation: |  |  |
| Sequence generation | 16a | Method of generating the allocation sequence (eg, computer-generated random numbers), and list of any factors for stratification. To reduce predictability of a random sequence, details of any planned restriction (eg, blocking) should be provided in a separate document that is unavailable to those who enrol participants or assign interventions   - Subjects will be arbitrarily assigned to the SGLT-2 inhibitor administration group or control group according to the random number table. |
| Allocation concealment mechanism | 16b | Mechanism of implementing the allocation sequence (eg, central telephone; sequentially numbered, opaque, sealed envelopes), describing any steps to conceal the sequence until interventions are assigned   - Not applicable (because our study is open blinded end-point study) |
| Implementation | 16c | Who will generate the allocation sequence, who will enrol participants, and who will assign participants to interventions   - Research manager and research officer |
| Blinding (masking) | 17a | Who will be blinded after assignment to interventions (eg, trial participants, care providers, outcome assessors, data analysts), and how   - Not applicable |
|  | 17b | If blinded, circumstances under which unblinding is permissible, and procedure for revealing a participant’s allocated intervention during the trial   - Not applicable |
| **Methods: Data collection, management, and analysis** | | |
| Data collection methods | 18a | Plans for assessment and collection of outcome, baseline, and other trial data, including any related processes to promote data quality (eg, duplicate measurements, training of assessors) and a description of study instruments (eg, questionnaires, laboratory tests) along with their reliability and validity, if known. Reference to where data collection forms can be found, if not in the protocol   - All of the data in this study consisted of bivariate of continuous numbers which can get according to standardized menuscript. (e.g. AF burden: evaluating time ratio of atrial fibrillation which detected at 24-hour Holter ECG more than 30 seconds, Disease-free survival rate, Left atrial size at echocardiography, NT-pro BNP, Symptom score (mEHRA), Quality of life (AFEQT) |
|  | 18b | Plans to promote participant retention and complete follow-up, including list of any outcome data to be collected for participants who discontinue or deviate from intervention protocols   - Participants meets our inclusion criteria – patient who have both atrial fibrillation and diabetes – need to regular hospital follow-up regardless of study, so we plan to promote participants to complete follow-up at their regular follow up for their underlying disease. |
| Data management | 19 | Plans for data entry, coding, security, and storage, including any related processes to promote data quality (eg, double data entry; range checks for data values). Reference to where details of data management procedures can be found, if not in the protocol   - The information of all subjects participating in this study will be managed in accordance with the relevant laws and regulations. It will be kept strictly confidential so as not to be exposed to third parties except related researchers, IRBs and research funding agencies, and will conduct research by coding or anonymizing so as not to be connected to the patient's personal information. In this study, the patient's address or contact information won’t be collected or managed separately, and the data file will not display the patient's personal identification number or patient's hospital ID. All data files must have a separate password to be managed by locking for security. |
| Statistical methods | 20a | Statistical methods for analysing primary and secondary outcomes. Reference to where other details of the statistical analysis plan can be found, if not in the protocol   - The study is powered to demonstrate the superior additive effect of SGLT-2 inhibitors over other hypoglycemic agents using the Chi square method. |
|  | 20b | Methods for any additional analyses (eg, subgroup and adjusted analyses)   - Atrial fibrillation recurrence rate, ratio of sinus rhythm on 24hr-holter EKG: Chi square test - Left atrial size, NT-pro BNP, quality of life (AFEQT) score on 12-month final follow-up: t-test - Disease-free survival and overall survival during the follow-up period are calculated using Kaplan-Meier method. |
|  | 20c | Definition of analysis population relating to protocol non-adherence (eg, as randomised analysis), and any statistical methods to handle missing data (eg, multiple imputation)   - Multiple imputation |
| **Methods: Monitoring** | | |
| Data monitoring | 21a | Composition of data monitoring committee (DMC); summary of its role and reporting structure; statement of whether it is independent from the sponsor and competing interests; and reference to where further details about its charter can be found, if not in the protocol. Alternatively, an explanation of why a DMC is not needed   - Instead of data monitoring committee, we have data monitoring plan performed by research manager and officer (checklist 21b) |
|  | 21b | Description of any interim analyses and stopping guidelines, including who will have access to these interim results and make the final decision to terminate the trial   - An interim analysis will be conducted at the time of 50% registration (based on the number of registered subjects for each group), and the items will be conducted according to the effective evaluation and statistical method. However, the timing of the interim analysis may vary depending on the patient's registration status. |
| Harms | 22 | Plans for collecting, assessing, reporting, and managing solicited and spontaneously reported adverse events and other unintended effects of trial interventions or trial conduct   - The evaluation of risks or side effects is planned to be conducted every three months according to the monitoring plan. |
| Auditing | 23 | Frequency and procedures for auditing trial conduct, if any, and whether the process will be independent from investigators and the sponsor   - Not applicable |
| Ethics and dissemination | | |
| Research ethics approval | 24 | Plans for seeking research ethics committee/institutional review board (REC/IRB) approval   - Approval of each institutional review board (IRB) of Ewha Womans University Mokdong Hospital and that of other medical centers participating in this study will be obtained before initiating the study. |
| Protocol amendments | 25 | Plans for communicating important protocol modifications (eg, changes to eligibility criteria, outcomes, analyses) to relevant parties (eg, investigators, REC/IRBs, trial participants, trial registries, journals, regulators)   - IRB routinely checked and re-approved annually study protocol, but important protocol modifications will be immediately reported to IRB. |
| Consent or assent | 26a | Who will obtain informed consent or assent from potential trial participants or authorised surrogates, and how (see Item 32)   - Research manager, co-researcher and research officer |
|  | 26b | Additional consent provisions for collection and use of participant data and biological specimens in ancillary studies, if applicable   - Not applicable |
| Confidentiality | 27 | How personal information about potential and enrolled participants will be collected, shared, and maintained in order to protect confidentiality before, during, and after the trial   - The information of all subjects participating in this study will be managed in accordance with the relevant laws and regulations. It will be kept strictly confidential so as not to be exposed to third parties except related researchers, IRBs and research funding agencies, and will conduct research by coding or anonymizing so as not to be connected to the patient's personal information. In this study, the patient's address or contact information won’t be collected or managed separately, and the data file will not display the patient's personal identification number or patient's hospital ID. All data files must have a separate password to be managed by locking for security. According to Article 15 of the Enforcement Rules of the Bioethics Act, research-related records shall be kept for three years from the end of the study, and documents or data files that have passed the storage period shall be destroyed in accordance with Article 16 of the Enforcement Decree of the Personal Information Protection Act. If storage is required for more than three years for follow-up research, records, or accumulation, a separate request will be made to the IRB to obtain permission to extend the storage period of data before the expiration of the validity period. |
| Declaration of interests | 28 | Financial and other competing interests for principal investigators for the overall trial and each study site   - The authors declare no competing financial interests. |
| Access to data | 29 | Statement of who will have access to the final trial dataset, and disclosure of contractual agreements that limit such access for investigators   - Research manager, co-researcher and research officer |
| Ancillary and post-trial care | 30 | Provisions, if any, for ancillary and post-trial care, and for compensation to those who suffer harm from trial participation   - Not applicable |
| Dissemination policy | 31a | Plans for investigators and sponsor to communicate trial results to participants, healthcare professionals, the public, and other relevant groups (eg, via publication, reporting in results databases, or other data sharing arrangements), including any publication restrictions   - Via Journal publication |
|  | 31b | Authorship eligibility guidelines and any intended use of professional writers   - Not applicable |
|  | 31c | Plans, if any, for granting public access to the full protocol, participant-level dataset, and statistical code   - Not applicable |
| Appendices |  |  |
| Informed consent materials | 32 | Model consent form and other related documentation given to participants and authorised surrogates   - Patient who meets the inclusion and exclusion criteria and voluntarily agreed to enroll the study will signed at documented written informed consent which reviewed and approved by IRB after sufficient explanation about this research process. |
| Biological specimens | 33 | Plans for collection, laboratory evaluation, and storage of biological specimens for genetic or molecular analysis in the current trial and for future use in ancillary studies, if applicable   - Not applicable |

*It is strongly recommended that this checklist be read in conjunction with the SPIRIT 2013 Explanation & Elaboration for important clarification on the items. Amendments to the protocol should be tracked and dated. The SPIRIT checklist is copyrighted by the SPIRIT Group under the Creative Commons “[Attribution-NonCommercial-NoDerivs 3.0 Unported](http://www.creativecommons.org/licenses/by-nc-nd/3.0/)” license.
