# Supplementary material for: Protocol of BEYOND trial: Clinical BEnefit of sodium-glucose cotransporter-2 (SGLT-2) inhibitors in rhYthm cONtrol of atrial fibrillation in patients with diabetes mellitus
Source: PLoS One. 2023 Jan 18;18(1):e0280359. doi: 10.1371/journal.pone.0280359 (PMC9847966; doi:10.1371/journal.pone.0280359)
Supplement: S2 File — (PDF) [file pone.0280359.s002.pdf]

연구계획서 *(version: 1.0)*

1. 연구의 제목 및 단계

1) 연구제목

(국문) 심방세동과 동반된 당뇨 환자에서의 SGLT-2 inhibitor 사용이 미치는 효과

(영문) The effect of SGLT-2 inhibitor in patient with atrial fibrillation and diabetes mellitus

2) 단계

- 해당없음

2. 연구의 실시기관명 및 주소

(주관)이대목동병원 순환기내과 박준범 교수 (서울시 양천구 안양천로 1071)

(공동)연세대학교신촌세브란스병원 순환기내과 박희남 교수 (서울시 서대문구 신촌동 연세로 50-1)

(공동)고려대학교안암병원 순환기내과 심재민 교수(서울시 성북구 안암동 고려대로 73)

(공동)한양대학교병원 순환기내과 박진규 교수 (서울시 성동구 사근동 왕십리로 222-1)

(공동)경희대학교병원 순환기 내과 이정명 교수 (서울시 동대문구 회기동 경희대로 23)

(공동)인하대학교병원 순환기내과 백용수 교수 (인천시 중구 신흥동 인항로 27)

(공동)이대서울병원 순환기내과 김동혁 교수 (서울시 강서구 발산1동 공항대로 260)

(공동)연세대학교용인세브란스병원 순환기내과 박제욱 교수 (경기도 용인시 기흥구 중동 동백죽전대로 363)

3. 연구책임자 및 연구담당자 등

1) 연구책임자 : 순환기내과 박준범 교수

2) 공동연구자: 내과 전공의 전보경

3) 연구담당자 : 순환기내과 정수미 연구간호사

4) 임상시험용 의약품 관리약사/임상시험용 의료기기 관리자 성명 및 직명 : 해당없음

4. 연구 의뢰기관

1) 연구 의뢰기관 명칭 : 해당없음

2) 모니터요원 직명 및 성명 : 해당없음

5. 연구비 지원기관

1) 연구비 지원기관 명칭

- 해당없음

6. 연구 대상 질환

- 심방세동과 동반된 당뇨 환자

7. 연구의 배경 및 목적

1) 연구의 배경

심방세동 (atrial fibrillation, AF) 은 가장 흔한 부정맥으로 연령에 따라 그 유병율이 증가하는 경향을 보인다. (1, 2) 미국에서의 유병율은 2010년 520만명으로 2030년에는 1210만명으로 더욱 증가할 것으로 보인다. (3) 한국에서의 유병율 또한 2006년 0.73%, 2015년 1.53% 로 꾸준한 증가 추세이다. (4) 심방세동의 치료에 대해서는 기존의 rate control을 통한 치료보다 항부정맥제 사용 및 전극도자절제술 등을 활용한 조기의 rhythm control을 통한 치료가 adverse cardiovascular event의 발생 확률을 낮춘다는 것이 최근 Early Treatment of Atrial Fibrillation for Stroke Prevention Trial (EAST-AFNET 4) 등을 통해 발표되고 있다. (5)

심방세동과 울혈성 심부전 (congestive heart failure, CHF) 은 고혈압, 당뇨, 허혈성 심질환 및 판막 질환과 같은 공통의 위험 인자를 공유하며 밀접하게 연관되어 있으나 그 병태생리학적 연관성에 대해서는 아직 더 많은 연구가 필요하다. (6-8) 현재 보고된 바 심부전은 심방충만압을 증가시키고 세포내 칼슘 수준을 변화시켜 심방세동의 위험을 높이는 것으로 알려져 있다. (9-12)

신장의 glucose 재흡수를 감소시켜 당뇨 환자의 고혈당을 조절하는 Sodium-glucose cotransporter 2 (SGLT2) inhibitor에 속하는 dapagliflozin 및 empagliflozin 은 Dapagliflozin and Prevention of Adverse Outcomes in Heart Failure (DAPA-HF) trial 및 The Empagliflozin Outcome Trial in Patients with Chronic Heart Failure and Reduced Ejection Fraction (EMPEROR-Reduced) trial을 비롯한 연구들에서 당뇨의 존재 유무에 관계없이 심부전의 악화 및 심혈관 질환으로 인한 사망률을 유의하게 감소시키는 것으로 보고되고 있다. (13, 14)

2) 연구 가설 및 목적

1. 연구의 목적

- Primary outcome

  1. 항부정맥제 사용 1년까지의 심방세동 재발률 (AF recurrence) 을 평가한다.

  2. 항부정맥제 사용 중 재발하여 전극도자 절제술을 시행 받은 환자의 경우 절제술 후 1년까지의 심방세동 재발률 (AF recurrence) 을 평가한다.

- Secondary outcome: 항부정맥제 사용 또는 전극도자절제술 후 1년까지의 리듬 조절 결과(AF free survival, AF burden) 및 전극도자절제술 시행률을 비교한다.

- Other Secondary outcome: AF free survival, 심초음파 상의 Left atrial size, NT-pro BNP, symptome score (mEHRA), quality of life (AFEQT)를 평가한다.

2. 연구의 가설

본 연구에서는 심부전에서 유의한 효과를 보이는 SGLT2 inhibitor 이 심부전과 당뇨를 비롯한 많은 수의 위험요인 및 병태생리학적 기전을 공유하는 심방세동에 긍정적 효과를 미칠 것으로 보고 그 효과를 평가한다.

8. 임상시험용 의약품 및 의료기기 정보 및 관리

-해당없음

9. 연구대상자의 선정기준, 제외기준, 목표한 대상자 수 및 산출근거

1) 선정기준

1. 20세 이상 80세 미만의 성인
2. 최근 1년 이내에 제2형 당뇨병을 처음으로 진단받은 환자 (당화혈색소 (HbA1c) 6.5% 이상) 중 아래 기준에 해당하는 환자
   - 경구 약제를 복용하지 않고 있는 경우 HbA1c 7.5% 이상
   - 경구 혈당강하제(metformin 단독 또는 2제/3제 요법)를 3개월 이상 사용한 경우 HbA1c 7.0% 이상
3. 최근 1년 이내에 EKG 상에서 확인된 심방세동을 진단받은 환자

2) 제외기준

1. 20세 미만 또는 80세 이상
2. 지난 2달 사이 다른 임상연구에 등록된 환자
3. 1년 이내의 기대여명을 가진 사람
4. 임산부, 수유부
5. 진단 당시 HbA1c 6.5% 미만 또는 12% 이상인 경우
6. 제1형 당뇨병
7. 인슐린을 사용하여 조절중인 제2형 당뇨병
8. 이미 SGLT-2 inhibitor를 사용하여 치료 중인 제2형 당뇨병
9. 최근 1년 사이 3차례 이상의 요로 감염 및 생식기 감염이 있었던 경우
10. 혈뇨가 확인된 경우
11. 수축기 혈압 > 180mmHg 또는 diastolic BP >100 mmHg
12. 수축기 혈압 < 95mmHg
13. 8주 이내에 acute cardiovascular event 가 있었던 경우 (stroke, acute coronary syndrome (ACS), revascularization, decompensated heart failure, sustained ventricular tachycardia, return of spontaneous circulation (ROSC)
14. 심각한 판막 질환이 있거나 인공 판막이 있는 경우

15. 신기능 장애 (eGFR-CKD-EPI <45ml/min/1.73m^2)

16. 임상적으로 확인된 간기능 장애

17. 조절되지 않는 갑상선 기능 이상

18. 최근 5년 이내에 암을 진단받은 경우

19. 최근 1달 이내에 경구 프레드니솔론 10mg/일 과 등가 또는 그 이상의 스테로이드 제제를 지속 사용한 경우

3) 중지 · 탈락기준

1. 생명을 위협하는 심각한 부작용의 발생
2. 본 연구와 관계없는, 예측하지 못한 의학적 소견 및 검사 결과
3. 환자가 강력히 치료 중단을 원할 때

4) 목표한 대상자 수 및 산출근거

- SGLT-2 inhibitor 투여군 352명, 대조군 352명을 목표 대상자 수로 선정하였다. (총 704명)

- 이는 G*Power를 사용하여 산출한 medium effect size, 유의수준 0.05, 검정력 80%의 이변량 통계 분석에 필요한 sample size (실험군, 대조군 각각 64명 이상 (총 128명 이상)) (15)에 항부정맥제 사용을 시작한 환자들에서 50% 에 이르는 전극도자절제술 시행률 (16, 17), SGLT-2 inhibitor 사용에 의해 기대되는 40% 정도의 AF 감소 효과 (18) 및 10% 의 환자 탈락률을 고려하여 산출한 결과이다.

- 본원에서는 200명의 대조군과 200명의 투여군으로 총 400명의 대상자를 목표로 한다.

10. 예상연구기간

: IRB 승인일 ~ 2026년 12월 31일

11. 연구방법

1) 구체적인 연구방법

1. Multicenter, prospective, 1:1 randomized, open blinded end-point study
2. 목표 대상자 선정: SGLT-2 inhibitor 투여군 352명, 대조군 352명 (총 704명)

3. 연구 참여 당시, 12개월 후 추적 검사: 12-lead EKG, 24hr-holter EKG, transthoracic echocardiography, Left atrial size, NT-pro BNP, quality of life (AFEQT)
4. 매 3개월 추적 관찰 시 시행 검사: 12-lead EKG, 24hr-holter EKG
5. 심방세동 환자에서 연구 참여 당시의 평가를 마친 후 바로 항부정맥제를 투여하며, 3개월 이후의 추적 관찰에서 재발할 경우 전극도자 절제술을 시행한다.
6. 항부정맥제 사용 이후 1년까지의 심방세동 재발 (AF recurrence) 및 AF burden을 평가한다.
7. 항부정맥제 사용 후 심방세동이 재발하여 전극도자 절제술을 시행 받은 환자의 경우 시행 후 1년까지의 심방세동 재발 (AF recurrence) 및 AF burden을 평가한다.
8. Left atrial size, NT-pro BNP, quality of life (AFEQT) 는 연구 참여 당시 및 1년 후 추적 종료 시 총 두차례 작성하여 평가한다. (additional secondary outcome)

2) 비교군 설정 및 무작위배정방법

피험자는 난수표에 따라 SGLT-2 inhibitor 투여군 또는 대조군으로 임의 배정된다. 대조군의 경우 SGLT-2를 제외한 다른 종류의 경구혈당강하제를 사용하여 혈당을 조절하게 된다.

3) 시험약 투여·사용량, 투여·사용방법, 병용요법, 대조약 사용시 그 선택사유

해당없음

4) 관찰항목 및 임상검사항목

A. 병명, 나이, 성별, 체중, 키, 흡연력, 음주력, 과거병력 및 약물 복용력, 혈액과 소변검사
B. 동반된 구조적 심장질환 양상 평가
C. 동반된 임상적 의미가 있는 질환 양상 평가
D. 영상학적 정량평가 (모든 검사는 연구 목적과 상관 없이 치료의 필요성에 의해 이루어지는 검사들로 구성되어 있으며 연구 목적으로 추가적인 영상학적 검사를 시행하지 않음)
- 심장초음파 검사: 심구혈률, 이완기능지표, 심방크기, 심실크기, 심방용적
E. 전기생리적 정량평가: 심내막 평균 전위차, 전도속도
F. 전극도자절제술에 대한 평가

5) 유효성 평가기준, 평가방법

SGLT-2 inhibitor 투여군 및 대조군에서

1. AF burden: 24-hour holter ECG에서 30초 이상 확인되는 심방세동의 시간 비율로 평가
2. Disease-free survival
3. Left atrial size
4. NT-pro BNP
5. Symptom score (mEHRA)
6. Quality of life (AFEQT)

을 비교하여 평가한다.

6) 기존치료와의 차별점

SGLT-2 inhibitor는 이미 당뇨환자에게서 metformin 과 함께 사용중인 경구혈당강하제 중 하나로 사용 여부에 따라 당뇨 환자의 치료 방침이 달라지지 않는다. 심방 세동 또한 표준 지침에 따라 치료를 진행하게 되며 다른 대체치료방법은 없다.

7) 연구대상자의 위험/이익 분석

본 연구는 심방세동 환자에게 SGLT-2 inhibitor 사용 여부를 제외한 부분에 있어 당뇨 및 심방세동의 표준적인 치료를 시행하며 임상경과를 관찰하게 되므로 본 연구에 참여함으로써 갖게 되는 추가적인 위험성이나 부작용은 없음.

SGLT-2 inhibitor 사용에 따른 대표적인 부작용인 요로감염 및 생식기 감염, 전해질 불균형, 기립성 저혈압 등에 대해서는 약제 투여 전 대상자 및 보호자에게 복약지도를 할 계획이며 연구 참여 후 추적관찰에서 스크리닝하여 해당 증상이 발생할 경우 투약을 중단할 계획임.

심방세동의 rhythm control 치료의 경우 가장 먼저 항부정맥제를 사용하게 되며 이것에 실패할 경우 (재발할 경우) catheter ablation을 시행하게 되는데, 사용하게 될 항부정맥제의 부작용에 대해서는 대상자나 보호자에게 복약지도를 할 계획임. Catheter ablation에 따른 시술 설명 및 위험성에 대해서는 현재 병원에서 사용하고 있는 별도의 설명문 및 동의서를 사용할 계획임. 하지만, 이러한 위험성은 현재의 표준 치료에서도 존재하는 위험성으로 본 연구에 의해 추가적으로 증가하는 위험성이나 부작용은 아님.

8) 부작용을 포함한 안전성의 평가기준, 평가방법 및 보고방법

A. SGLT-2 inhibitor는 이미 제 2형 당뇨 환자에서 경구혈당강하제로서 사용되고 있는 약으로 일반적으로 알려진 부작용 외에 추가적으로 본 연구를 진행함으로써 발생하는

부작용이나 안전성에는 해당사항이 없음.

B. 연구 등록 후 3개월, 6개월, 9개월, 12개월째 추적 관찰에서 환자 문진 및 신체 진찰을 통하여 투약중인 약제의 부작용 여부를 평가하며, 12-lead EKG, 24hr-holter EKG, AF symptom score (mEHRA) 를 통하여 AF burden 및 심방세동의 재발 유무를 평가함.

C. B항목을 진료시 외래기록지에 심전도와 함께 기록하게 됨.

D. 3개월 추적관찰시 대상자가 보고하는 모든 이상 사례들은 연 4회 연구담당자 간의 회의에서 보고 계획이며 상기 이상 반응이 SGLT-2 inhibitor (dapaglifozin, empagliflozin) 의 식품의약품안전처 고시문에 따른 인과관계를 배제할 수 없는 중대한 약물이상반응 (과민 반응, 비뇨생식기 감염, 요로결석, 전해질 불균형, 기립성 저혈압) 또는 인과관계와 상관 없는 중대한 이상사례에 해당한다고 판단될 경우 회의를 통해 참여 중단을 결정할 계획임.

9) 자료안전성 모니터링 계획(DSMP)

- 50% 등록 (각 군별 등록 대상자수 기준) 시점에 중간 분석을 실시할 계획이며 항목에 대해서는 아래에 기술할 유효성 평가 및 아래에 기술된 통계학적 방법에 따라 시행할 계획임. 위험성이나 부작용 등에 대한 평가는 11-8)번 항목의 모니터링에 따라 3개월마다 시행할 계획임. 하지만 환자의 등록 상황에 따라 중간 분석의 시기는 달라질 수 있음.

10) 자료 분석 및 통계분석방법

1. 연구의 유효성 평가 항목

- 심방세동 재발률 (AF recurrence)

- 24-hour holter EKG 및 AF symptom score (EHRA) 를 통한 AF burden 및 Disease-free survival rate

- Left atrial size, NT-pro BNP, symptome score (mEHRA), quality of life (AFEQT)

2. 통계분석 방법

- 심방세동 재발률, 24hr-holter EKG 상에서의 sinus rhythm의 비율, 12개월 최종 추적 관찰시의 Left atrial size, NT-pro BNP, quality of life (AFEQT) 점수를 산출하고 t-test로 비교

- 추적 기간 동안의 disease-free survival 및 전체 생존률을 Kaplan-Meier 법으로 계산

11) 연구 도식도

Flow chart of the trial

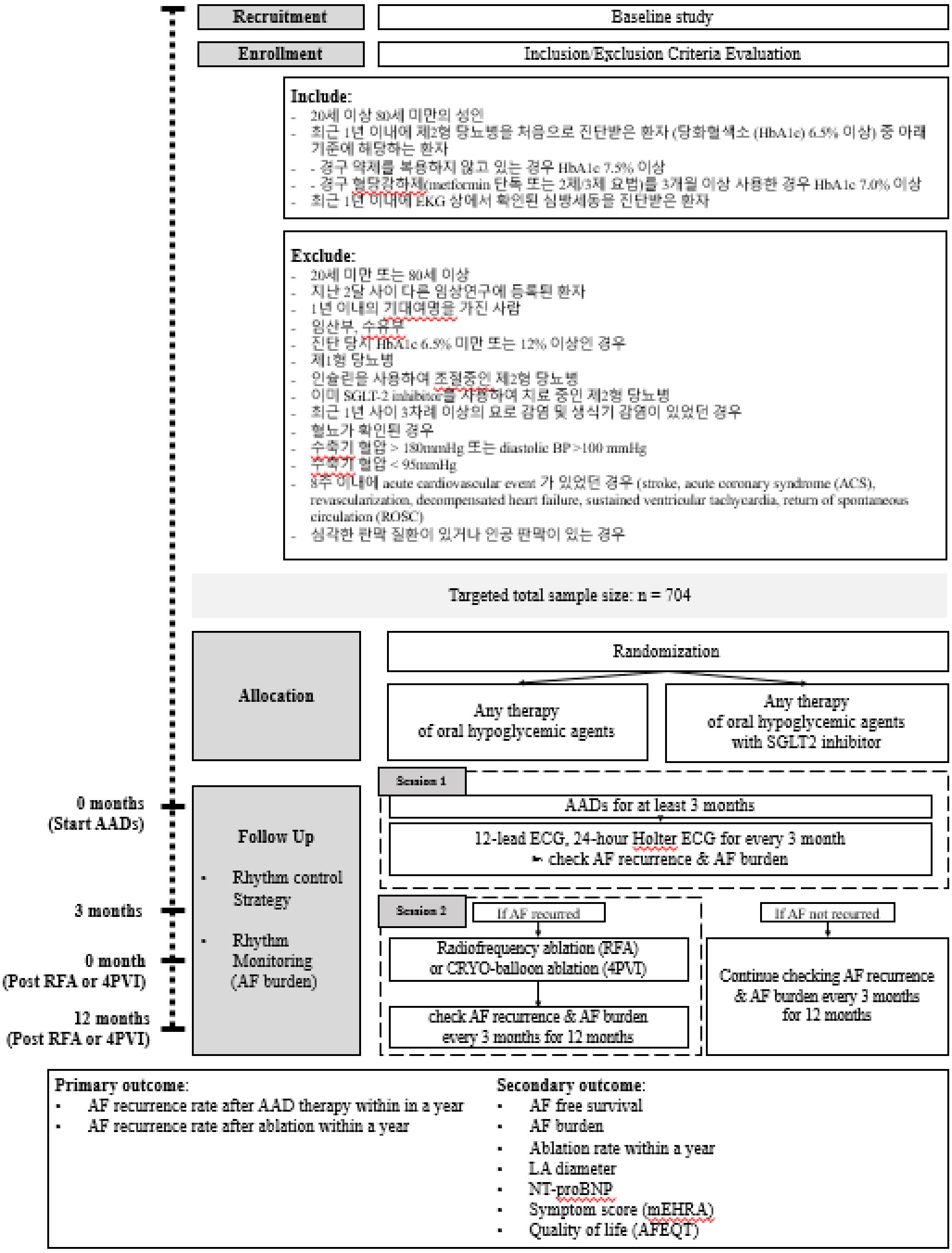

Abbreviations: DM, diabetes mellitus; AF, atrial fibrillation; EPI, epidemiology collaboration; AAD, Anti-arrhythmic drugs; ECG, electrocardiogram; RFA, radiofrequency ablation; 4PVI, 4 pulmonary vein isolation; LA, Left atrium; NT-proBNP, N-terminal pro-B-type natriuretic peptide

12) 연구수행 계획표(일정표)

수행일정	세부 추진 일정 (65 개월)											
	2021년8월 -9월		2021년10월-2022년12월			2023년1월 -12월		2024년1월-2025년12월			2026년1월 -12월	
연구계획 및 IRB승인	■	■										
대상자 모집 및 연구절차 수행			■	■	■	■	■					
추적조사						■	■	■	■	■		
자료분석 및 결과보고											■	■

13) 연구 대상자의 당뇨 관리 계획

2021 ADA (American Diabetes Associaton) guideline에 따르면 특별한 금기가 없을 경우 당뇨병의 초치료약제로는 metformin을 권고하며, metformin 사용해도 효과가 없거나 initial HbA1c 가 7.5% 이상으로 높을 경우 metformin 과 함께 sulfonylurea, meglitinide, alpha-glucosidase inhibitor, thiazolidinedione, DPP-4 inhibitor, SGLT-2 inhibitor 계열의 약제들을 추가하는 2제 요법을 시행할 것을 권고하고 있다.

이에 연구 등록 여부와 상관없이 당뇨 치료 지침에 따른 표준 치료를 수행하기 위해 inclusion criteria 의 당뇨 항목을 아래와 같이 제한하였으며, 이에 따른 실험군 및 대조군의 당뇨 관리 계획은 다음과 같다.

실험군 (SGLT-2 inhibitor 투약군)

경구 약제를 복용하지 않는 HbA1c 7.5% 이상의 환자

➔ 초치료로 metformin 과 SGLT-2 inhibitor 2제 요법을 시작한다.

경구 metformin 을 3개월 이상 사용한 환자 중 HbA1c 7.0% 이상

➔ 기존에 사용 중인 metformin에 SGLT-2 inhibitor를 추가한다.

2제 요법 또는 3제 요법을 시행중인 환자

➔ SGLT-2 inhibitor를 포함한 2제 또는 3제 요법으로 유지 또는 변경한다.

두 경우 모두 3개월에 한번 HbA1c 를 추적할 계획이며, SGLT-2 inhibitor 사용에도 혈당 조절이 불량할 경우 sulfonylurea, meglitinide, alpha-glucosidase inhibitor, thiazolidinedione, DPP-4 inhibitor 와 같은 다른 약제를 추가하는 3제 요법을 시행한다.

대조군

경구 약제를 복용하지 않는 HbA1c 7.5% 이상의 환자

➔ 초치료로 metformin 과 함께 sulfonylurea, meglitinide, alpha-glucosidase inhibitor, thiazolidinedione, DPP-4 inhibitor 계열 약제 중 하나를 포함한 2제 요법을 시작한다.

경구 metformin 을 3개월 이상 사용한 환자 중 HbA1c 7.0% 이상

➔ 기존에 사용 중인 metformin에 sulfonylurea, meglitinide, alpha-glucosidase inhibitor, thiazolidinedione 또는 DPP-4 inhibitor 계열 약제 중 하나를 추가한다.

2제 요법 또는 3제 요법을 시행중인 환자

➔ SGLT-2 inhibitor를 포함하지 않는 2제 또는 3제 요법으로 유지 또는 변경한다.

대조군의 경우에도 3개월에 한번 HbA1c 를 추적할 계획이며 필요에 따라 SGLT-2 inhibitor 외 다른 약제를 추가하는 3제 요법을 시행한다.

(* SGLT-2 inhibitor 약물과 그 외 다른 경구 혈당강하제 정보는 별첨1. 당뇨약물정보 참조.)

12. 연구대상자의 안전보호를 위한 대책

1) 연구의 윤리성 확보를 위한 기본 방안

본 연구는 임상시험심사위원회/기관생명윤리심의위원회에서 본 연구의 윤리적, 법적 요건을 충분히 검토하여 승인한 임상시험계획서에 의하여 시행되며, 또한 시험의 전 과정에 걸쳐 임상시험 관리기준 및 헬싱키 선언(2013년 개정)의 근본정신을 준수하게 될 것이다. 또한 본 연구는 ICH-GCP 및 KGCP를 준수하며 IRB 승인 후 연구를 수행할 것이다. 본 연구 도중에 연구대상자에 인권에 침해가 발생했을 경우 임상시험 심사위원회/기관생명윤리심의위원회에 통보될 것이다. 연구대상자의 신원을 파악할 수 있는 정보는 연구자에 의해 비밀로 보장될 것이며, 이니셜과 코드화 된 연구대상자 식별정보로 연구 자료가 기록될 것이다. 또한 연구대상자 및 연구정보는 접근 제한된 컴퓨터에 저장될 것이며 연구대상자의 신상을 보호하기 위해 독립된 공간에서 연구에 대해 설명하고 동의서를 취득할 것이다. 임상시험의 결과가 출판될 경우에도 연구대상자 신상정보는 비밀상태로 유지될 것이다

2) 연구대상자의 동의 과정

- 연구대상자에게 설명하고 동의를 취득할 연구자: 연구책임자, 연구담당자
- 동의를 제공할 자: 연구대상자 또는 대리인

- 연구설명과정과 동의 취득과정의 설명 시간: 30분 (검사실/진료실 내원 시 취득)
- 강제 또는 부당한 영향의 가능성을 최소화시킬 방법: 연구 참여는 환자 본인의 자발적인 의사에 의해서만 가능하며, 만일 참여를 원하지 않더라도 추후의 치료 과정에는 어떤 영향도 미치지 않을 것임을 설명함.
- 연구 설명과정과 동의 취득 과정에서 연구자가 사용하는 언어: 한국어
- 연구대상자 또는 대리인이 이해할 수 있는 언어: 한국어

3) 연구대상자의 보상방안

해당없음

4) 연구대상자의 개인정보보호 방안

본 연구에 참여하는 모든 대상자의 정보는 해당 법률과 규정에 따라 관리될 것이다. 관련 연구자와 IRB와 연구비지원 기관을 제외한 제 3자에 노출되지 않도록 철저히 기밀을 유지하여 보관하며, 환자의 개인정보와 연결되지 않도록 코드화 또는 익명화하여 연구를 진행할 것이다. 본 연구에서는 환자의 주소나 연락처 정보를 별도로 수집하거나 관리하지 않으며, 자료를 분석하는 데이터 파일에는 환자의 개인 신상을 확인할 수 있는 주민등록번호 또는 환자의 병록 번호를 표시하지 않을 것이다. 모든 데이터 파일에는 보안을 위한 잠금 설정을 하여 별도로 관리하는 암호가 있어야만 파일의 내용에 접근할 수 있도록 한다. 생명윤리법 시행 규칙 제 15조에 따라 연구 관련 기록은 연구가 종료된 시점부터 3년간 보관하며, 보관 기간이 지난 문서나 데이터 파일은 개인정보보호법 시행령 제 16조에 따라 파기하도록 한다. 후속 연구나 기록, 축적 등을 위해 3년 이상 보관이 필요한 경우라면 유효 기간이 경과하기 전 IRB에 자료의 보관 기한을 연장하는 것을 허가 받기 위한 별도의 요청을 하기로 한다.

5) 취약한 연구대상자를 포함하는 경우 추가적인 보호조치 방안

해당없음.

13. 인체유래물, 유전정보 등 수집 시 관리·보관·폐기 방안

해당없음

14. 참고문헌

1. Benjamin EJ WP, D'Agostino RB, Silbershatz H, Kannel WB, Levy D. Impact of atrial fibrillation on the risk of death: the Framingham Heart Study. Circulation. 1998;98(10):946-52.
2. January CT, Wann LS, Alpert JS, Calkins H, Cigarroa JE, Cleveland JC, Jr., et al. 2014 AHA/ACC/HRS guideline for the management of patients with atrial fibrillation: a report of the American College of Cardiology/American Heart Association Task Force on practice guidelines and the Heart Rhythm Society. Circulation. 2014;130(23):e199-267.
3. Colilla S, Crow A, Petkun W, Singer DE, Simon T, Liu X. Estimates of current and future incidence and prevalence of atrial fibrillation in the U.S. adult population. Am J Cardiol. 2013;112(8):1142-7.
4. Kim D, Yang PS, Jang E, Yu HT, Kim TH, Uhm JS, et al. 10-year nationwide trends of the incidence, prevalence, and adverse outcomes of non-valvular atrial fibrillation nationwide health insurance data covering the entire Korean population. Am Heart J. 2018;202:20-6.
5. Kirchhof P, Camm AJ, Goette A, Brandes A, Eckardt L, Elvan A, et al. Early Rhythm-Control Therapy in Patients with Atrial Fibrillation. N Engl J Med. 2020;383(14):1305-16.
6. Kareti KR, Chiong JR, Hsu SS, Miller AB. Congestive heart failure and atrial fibrillation: rhythm versus rate control. J Card Fail. 2005;11(3):164-72.
7. Heist EK, Ruskin JN. Atrial fibrillation and congestive heart failure: risk factors, mechanisms, and treatment. Prog Cardiovasc Dis. 2006;48(4):256-69.
8. Anter E, Jessup M, Callans DJ. Atrial fibrillation and heart failure: treatment considerations for a dual epidemic. Circulation. 2009;119(18):2516-25.
9. SOLTI F, VECSEY T, KÉKESI V, JUHÁSZ-NAGY A. The effect of atrial dilatation on the genesis of atrial arrhythmias. Cardiovascular Research. 1989;23(10):882-6.
10. Frank Bode AK, Raymond L. Woosley, and Michael R. Franz. Gadolinium Decreases Stretch-Induced Vulnerability to Atrial Fibrillation. Circulation. 2000;101(18):2200-5.
11. Dirk J. Beuckelmann MMN, MD; and Erland Erdmann, MD. Intracellular Calcium Handling in Isolated Ventricular Myocytes From Patients With Terminal Heart Failure. Circulation. 1992;85(3):1046-55.
12. Ohkusa T, Ueyama T, Yamada J, Yano M, Fujumura Y, Esato K, et al. Alterations in cardiac sarcoplasmic reticulum Ca2+regulatory proteins in the atrial tissue of patients with chronic atrial fibrillation. Journal of the American College of Cardiology. 1999;34(1):255-63.
13. McMurray JJV, Solomon SD, Inzucchi SE, Kober L, Kosiborod MN, Martinez FA, et al. Dapagliflozin in Patients with Heart Failure and Reduced Ejection Fraction. N Engl J Med. 2019;381(21):1995-2008.
14. Packer M, Anker SD, Butler J, Filippatos G, Pocock SJ, Carson P, et al. Cardiovascular and Renal Outcomes with Empagliflozin in Heart Failure. N Engl J Med. 2020;383(15):1413-24.

15. Faul F, Erdfelder E, Buchner A, Lang A-G. Statistical power analyses using G* Power 3.1: Tests for correlation and regression analyses. Behavior research methods. 2009;41(4):1149-60.
16. Andrade JG, Deyell MW, Verma A, Macle L, Champagne J, Leong-Sit P, et al. Association of atrial fibrillation episode duration with arrhythmia recurrence following ablation: a secondary analysis of a randomized clinical trial. JAMA network open. 2020;3(7):e208748-e.
17. Fernandes GC, Fernandes A, Cardoso R, Penalver J, Knijnik L, Mitrani RD, et al. Association of SGLT2 inhibitors with arrhythmias and sudden cardiac death in patients with type 2 diabetes or heart failure: A meta-analysis of 34 randomized controlled trials. Heart rhythm. 2021.
18. Andrade JG, Wells GA, Deyell MW, Bennett M, Essebag V, Champagne J, et al. Cryoablation or drug therapy for initial treatment of atrial fibrillation. New England Journal of Medicine. 2021;384(4):305-15.
