# Supplementary material for: Protocol of BEYOND trial: Clinical BEnefit of sodium-glucose cotransporter-2 (SGLT-2) inhibitors in rhYthm cONtrol of atrial fibrillation in patients with diabetes mellitus
Source: PLoS One. 2023 Jan 18;18(1):e0280359. doi: 10.1371/journal.pone.0280359 (PMC9847966; doi:10.1371/journal.pone.0280359)
Supplement: S3 File — (DOCX) [file pone.0280359.s003.docx]

**Research Plan** *(version: 1.0)*

1. **Subjects and steps of the study**
2. **Subjects**

**(Korean) 심방세동과 동반된 당뇨 환자에서의 SGLT-2 inhibitor 사용이 미치는 효과**

**(English) The effect of SGLT-2 inhibitor in patient with atrial fibrillation and diabetes mellitus**

1. **Steps**

- Not applicable

1. **Name and address of the research organization**

(main) Junbeom Park, a professor of cardiology at Ewha Womans University Mokdong Hospital (1071, Anyangcheon-ro, Yangcheon-gu, Seoul)

(co-work) Hui-Nam Pak, a professor of cardiology at Yonsei University's Sinchon Severance Hospital (50-1 Yonsei-ro, Sinchon-dong, Seodaemun-gu, Seoul)

(co-work) Jaemin Shim, a professor of cardiology at Korea University Anam Hospital (73, Korea University, Anam-dong, Seongbuk-gu, Seoul)

(co-work) Jin-Kyu Park, a professor of cardiology at Hanyang University Hospital (222-1 Wangsimni-ro, Sagun-dong, Seongdong-gu, Seoul)

(co-work) Jung Myung Lee, a professor of cardiology at Kyung Hee University Hospital (23, Kyung Hee-daero, Hoegi-dong, Dongdaemun-gu, Seoul)

(co-work) Yong-Soo Baek, a professor of cardiology at Inha University Hospital (27, Sinheung-dong, Jung-gu, Incheon)

(co-work) Dong Hyeouk Kim, a professor of cardiology at Ewha Womans University Seoul Hospital (260 Airport-daero, Balsan 1-dong, Gangseo-gu, Seoul)

(co-work) Park Je-wook, a professor of cardiology at Yonsei University's Yongin Severance Hospital (363, Dongdong Dongbaekjukjeon-daero, Giheung-gu, Yongin-si, Gyeonggi-do)

1. **Research manager and participant, etc.**
2. Research Manager: Junbeom Park, professor of cardiology
3. Co-researcher: Bo Kyung Jeon, fellow of cardiology
4. Research Officer: Sumi Jung, research nurse of cardiology
5. Clinical Pharmaceutical Management Pharmacist / Clinical Medical Device Manager: Not Applicable
6. **Commissioning organization**
7. **Name of the research request organization:** Not applicable
8. **Name of monitoring personnel:** Not applicable
9. **Research funding organization**
10. **Name of research funding organization**

- Not applicable

1. **Diseases of the Study**

- DM patient with atrial fibrillation

1. **Background & Objectives**
2. **Background**

Atrial fibrillation (AF) is a common cause of cardiac arrhythmia, and its prevalence increases with age. (1, 2) In the United States alone, the prevalence of AF is expected to increase to 12.1 million in 2030 from 5.2 million in 2010. (3) The prevalence in Korea is also steadily increasing to 0.73% in 2006 and 1.53% in 2015. (4) As for the treatment of atrial fibrillation, according to recent studies including Early Treatment of Atrial Fibrillation for Stroke Prevention Trial (EAST-AFNET 4), early rhythm-control treatment with antiarrhythmic drug and catheter ablation can reduces the incidence of adverse cardiovascular events compared to conventional rate control. (5) AF and congestive heart failure (CHF) are commonly encountered disease entities that share common risk factors such as hypertension, DM, ischemic heart disease, and valvular heart disease, but their pathophysiologic relationship are still unknown. (6-8) It has been suggested that heart failure (HF) increases the risk of AF by elevating atrial filling pressures and inducing alterations in intracellular calcium. (9-12) The Dapagliflozin and Prevention of Adverse Outcomes in Heart Failure (DAPA-HF) trial and The Empagliflozin Outcome Trial in Patients with Chronic Heart Failure and Reduced Ejection Fraction (EMPEROR-Reduced) trial revealed that two sodium–glucose cotransporter 2 (SGLT-2) inhibitors, dapagliflozin and empagliflozin, reduce the risk of cardiovascular death or HF exacerbation, regardless of the presence or absence of DM. (13, 14)

1. **Research Hypothesis and Objective**

1. Objective

- Primary outcome

1. Evaluating recurrence rate of atrial fibrillation (AF recurrence) up to one year after using antiarrhythmic drugs

2. In patients who have undergone catheter ablation due to the recurrence during the use of antiarrhythmic drugs, evaluating recurrence rate of atrial fibrillation (AF recurrence) up to one year after ablation.

- Secondary outcome: The results of rhythm control (AF free survival, AF burden) and rate of ablation up to 1 year after antiarrhythmic drug usage or ablation.

- Other Secondary outcome: AF free survival, Left atrial size at echocardiography, NT-pro BNP, symptome score (mEHRA), quality of life (AFEQT)

2. Hypothesis

This trial assume that SGLT-2 inhibitors have an advantageous influence on the rhythm control of AF and, hence, reduce the adverse outcomes of AF.

1. **Information and management of pharmaceuticals and medical devices for clinical trials**

- Not applicable

1. **Inclusion criteria, exclusion criteria, target number of subjects and basis for calculation**
2. **Inclusion Criteria**

1. 20 ≤ age < 80

2. Among the patients who are diagnosed with type 2 diabetes within one year (more than 6.5% of glycated hemoglobin (HbA1c)), those who meet the following criteria

- HbA1c ≥ 7.5%, if patient have no oral DM medication

- HbA1c ≥ 7.0%, if patient already have oral hypoglycemic agents (metformin alone or double/triple-agent therapy) more than 3 months

3. Patients who are diagnosed with atrial fibrillation identified on EKG within one year

1. **Exclusion Criteria**

1. Under 20 years old or over 80 years

2. Subject for another clinical trial within the past 2 months

3. Any disease that limits life expectancy to under 1 year

4. Pregnant/Lactating women

5. HbA1c ≥ 12% or HbA1c < 6.5% at diagnosis

6. Diagnosis of Type 1 DM

7. Type 2 DM treated by recombinant insulin

8. Previous treatment with any SGLT-2 inhibitor

9. Chronic cystitis and/or recurrent genitourinary tract infections (3 or more in the last year)

10. Unexplained hematuria at baseline study

11. Systolic BP > 180 mmHg or diastolic BP > 100 mmHg at baseline study

12. Systolic BP < 95 mmHg at baseline study

13. Acute cardiovascular event [e.g., stroke, acute coronary syndrome (ACS), revascularization, decompensated HF, sustained ventricular tachycardia, return of spontaneous circulation (ROSC)] <8 weeks prior to baseline study

14. Severe valvular disease or have prosthetic valve

15. Renal dysfunction (eGFR-CKD-EPI <45ml/min/1.73m^2^)

16. Clinically profound hepatic dysfunction

17. Clinically uncontrolled thyroid dysfunction

18. History of any malignancy within 5 years

19. Treatment with chronic oral steroid (>30 consecutive days) at a dose equivalent to oral prednisolone ≥ 10 mg/d, within the past 1 month

1. **Suspension and drop-out criteria**

1. Occurrence of serious life-threatening side effects

2. Unexpected medical and laboratory findings unrelated to study

3. When the patient strongly wishes to discontinue treatment

1. **Target number of subjects and basis for calculation**

- 352 people as SGLT-2 inhibitor administration group and 352 people as control group were selected as the target number of subjects. (A total of 704 people)
- This sample size is initially calculated using G*Power program (bivariate statistical analysis, p = 0.05, power 80%), then consider the ratio of reduction (40%) in AF after a year of treatment by SGLT-2 inhibitor, ablation (50%) of AAD users and follow-up loss (10%). (15-18)
- In our hospital, a total of 400 subjects are targeted with 200 control groups and 200 administration groups.

1. **Estimated Research Period**

: Date of IRB approval ~ 2026/Dec/31

1. **Methods**
2. **Detailed Methods**

1. Multicenter, prospective, 1:1 randomized, open blinded end-point study

2. Target sample size: 352 people as SGLT-2 inhibitor administration group, 352 people as control group (Total 704 people)

3. Examination which checked time of enrollment & after 12 months: 12-lead EKG, 24hr-holter EKG, transthoracic echocardiography, Left atrial size, NT-pro BNP, quality of life (AFEQT)

4. Examination which checked every 3-month follow-up: 12-lead EKG, 24hr-holter EKG

5. Antiarrhythmic drug is administered immediately after completing the evaluation at the time of enrollment in the study in patients with atrial fibrillation, and ablation will be performed if recurrence occurs in follow-up after 3 months.

6. Atrial fibrillation recurrence (AF recurrence) and AF burden up to 1 year after use of antiarrhythmic drug will be evaluated.

7. For patients who underwent ablation due to recurrence of atrial fibrillation after use of antiarrhythmic drugs, AF recurrence and AF burden up to 1 year after ablation are evaluated.

8. Left atrial size, NT-pro BNP, and quality of life (AFEQT) are evaluated twice at the time of study enrollment and at the end of the follow-up one year later. (additional secondary outcome)

1. **Control Group Configuration and Randomized Allocation Method**

Subjects will be arbitrarily assigned to the SGLT-2 inhibitor administration group or control group according to the random number table. In the case of the control group, serum glucose level will be controlled using other types of oral glucose lowering agents except SGLT-2 inhibitor.

1. **Test drug administration dose, administration method, combination method, and reasons for choice when using a control drug.**

Not applicable

1. **Observation and clinical examination items**

A. Name of the disease, age, gender, weight, height, smoking history, alcohol history, medical history and blood and urine tests

B. Evaluating combined structural heart disease patterns

C. Evaluating accompanying clinically significant disease profile

D. Quantitative imaging evaluation (all tests will be performed for the need for treatment regardless of the purpose of the study, and no additional imaging tests will be performed only for study)

- echocardiography: EF, relaxation abnormality, atrial size, ventricular size, atrial volume

E. EP study: average endocardial potential difference, conduction velocity

F. Evaluation for ablation

1. **Criteria & methods of evaluating significancy**

Comparing

1. AF burden: evaluating time ratio of atrial fibrillation which detected at 24-hour Holter ECG more than 30 seconds
2. Disease-free survival
3. Left atrial size
4. NT-pro BNP
5. Symptom score (mEHRA)
6. Quality of life (AFEQT)

at SGLT-2 inhibitor administration group & control group

1. **Difference from conventional treatment**

SGLT-2 inhibitor is one of the oral hypoglycemic agents which already used with metformin in diabetic patients, so treatment policy of diabetic patients does not change depending on whether SGLT-2 inhibitor is used or not. Atrial fibrillation will be also treated according to standard guidelines, and there is no other alternative treatments.

1. **Risk/benefit analysis of study subjects**

In this study, there are no additional risks or side effects from participating in this study because standard treatment of diabetes and atrial fibrillation is performed in patients with atrial fibrillation except for the use of SGLT-2 inhibitor.

For genitourinary infections, electrolyte imbalance and orthostatic hypotension, which are representative side effects of the use of SGLT-2 inhibitor, we plan to provide medication guidance to subjects and their guardians before drug administration and to stop administration if such side effects are detected at follow-up.

In the case of rhythm control treatment of atrial fibrillation, antiarrhythmic drugs will be used first, then catheter ablation is performed (in case of recurrence), and the information of side effects of the antiarrhythmic drugs will be given to subjects or guardians.

For the explanation and risk of the procedure of Catheter ablation, we plan to use separate explanations and consent forms currently used in our hospitals. However, this risk is already present in the current standard treatment so is not an additional risk or side effect increased by this study.

1. **Safety evaluation criteria, methods, and reporting methods including side effects**

A. SGLT-2 inhibitor is not applicable to the side effects arising from this study because it is commonly known as oral hypoglycemic drugs already used in type 2 diabetic patients.

B. Follow-up at 3, 6, 9, and 12 months after registration evaluates the side effects of drugs through patient questioning and physical examination, and evaluates AF burden and atrial fibrillation recurrence through 12-lead EKG, 24hr-holter EKG, and AF symscore (meHRA).

C. Item B will be recorded in the outpatient record sheet along with an electrocardiogram.

D. All adverse events reported by the subjects during a 3-month follow-up will be reported at meetings between researchers four times a year, and interruption of participation will be decided if above adverse reaction is significant side effect of SGLT-2 inhibitor (dapaglifozin, empagliflozin) which informed by Ministry of Food and Drug Safety or serious adverse events unrelated to causality.

1. **Data safety monitoring plan (DSMP)**

- An interim analysis will be conducted at the time of 50% registration (based on the number of registered subjects for each group), and the items will be conducted according to the effective evaluation and statistical method described below. The evaluation of risks or side effects is planned to be conducted every three months according to the monitoring plan mentioned at 11-8. However, the timing of the interim analysis may vary depending on the patient's registration status.

1. **Data analysis and statistical analysis methods**

1. Efficacy of research evaluation item

- AF recurrence

- AF burden and Disease-free survival rate according to 24-hour holter EKG and AF symptom score (EHRA)

- Left atrial size, NT-pro BNP, symptome score (mEHRA), quality of life (AFEQT)

2. Statistical analysis method

- Atrial fibrillation recurrence rate, ratio of sinus rhythm on 24hr-holter EKG, Left atrial size, NT-pro BNP, quality of life (AFEQT) score on 12-month final follow-up -> compare with t-test

- Disease-free survival and overall survival during the follow-up period are calculated using Kaplan-Meier method.

1. **Schematic Diagram of Research**


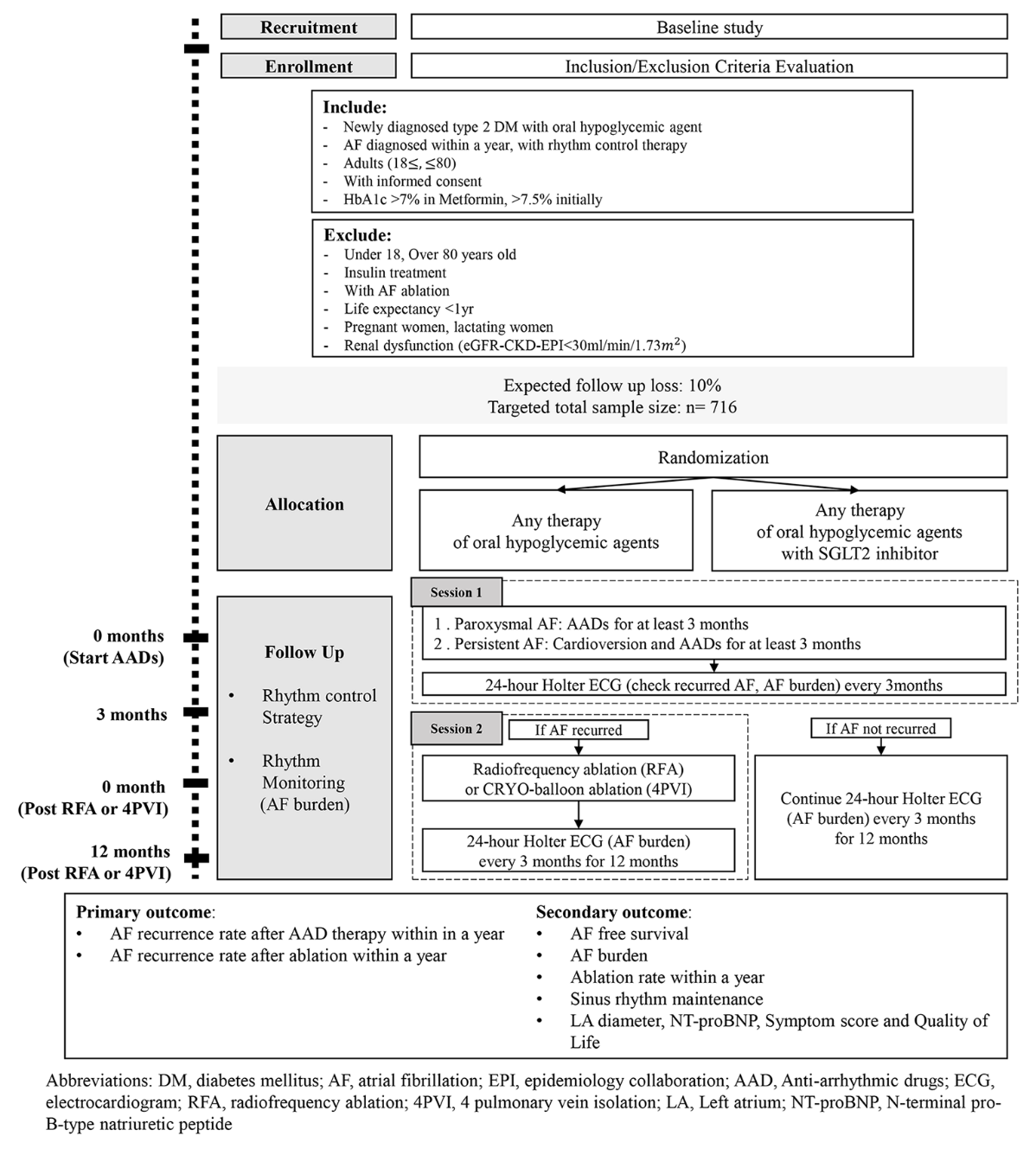


1. **Research Action Plan (Schedule)**

| schedule of performance | detailed implementation schedule (65 months) | | | | | | | | | | | |
| --- | --- | --- | --- | --- | --- | --- | --- | --- | --- | --- | --- | --- |
|  | 2021/08-09 | | 2021/10-2022/12 | | | 2023/01-12 | | 2024/01-2025/12 | | | 2026/01-12 | |
| Research Plan and IRB Approval | ■ | ■ |  |  |  |  |  |  |  |  |  |  |
| Recruitment of subjects and conduct of research procedures |  |  | ■ | ■ | ■ | ■ | ■ |  |  |  |  |  |
| Follow-up |  |  |  |  |  | ■ | ■ | ■ | ■ | ■ |  |  |
| Data analysis and reporting |  |  |  |  |  |  |  |  |  |  | ■ | ■ |

**13) Study subjects' diabetes management plan**

According to 2021 ADA (American Diabetes Association) guidelines, metformin is recommended as a first-line drug for diabetes if there is no specific contraindication, and two-drug therapy which combined metformin and other drugs such as sulfonylurea, meglitinide, alpha-glucosidase inhibitor, thiazolidinedione, DPP-4 inhibitor and SGLT-2 inhibitor is recommended if metformin is not effective or if initial HbA1c is higher than 7.5%.

Therefore, in order to perform standard treatment according to the ADA guidelines regardless of whether or not study registration, the diabetes of inclusion criteria was limited as follows, and the diabetes management plan of the experimental group and control group is as follows;

**Experimental group (SGLT-2 inhibitor administration group)**

HbA1c ≥7.5% of patients who are not taking oral medications

- Begin two-drug therapy - metformin and SGLT-2 inhibitor - as a first-line therapy

HbA1c 7.0% or more of patients who have used oral metformin for more than 3 months

- SGLT-2 inhibitor will be added to the previously used metformin.

Patients already taking two-drug or three-drug therapy

- Maintained or changed to a two or three-drug therapy containing SGLT-2 inhibitor.

In both cases, HbA1c is planned to be tracked every three months, and if blood sugar control is poor even with the use of the SGLT-2 inhibitor, three-drug therapy that adding such as sulfonylurea, meglitinide, alpha-glucosidase inhibitor, thiazolidine and DPP-4 inhibitor will be applied.

**Control group**

HbA1c ≥7.5% of patients who are not taking oral medications

- Begin two-drug therapy - metformin and one of the sulfonylurea, meglitinide, alpha-glucosidase inhibitor, thiazolidinedione, DPP-4 inhibitor - as a first-line therapy

HbA1c 7.0% or more of patients who have used oral metformin for more than 3 months

- Sulfonylurea, meglitinide, alpha-glucosidase inhibitor, thiazolidinedione or DPP-4 inhibitor will be added to the previously used metformin.

Patients already taking two-drug or three-drug therapy

- Maintained or changed to a two or three-drug therapy containing other than SGLT-2 inhibitor.

In the case of the control group, HbA1c is also planned to be tracked every three months, and if necessary, a three-drug therapy is performed to add drugs other than SGLT-2 inhibitor.

1. **Plans to protect the safety of the study subjects**
2. **Basic methods to ensure the ethics of research**

This study is conducted in accordance with a clinical trial plan approved by the Clinical Trial Review Committee/Institutional Review Board, and will comply with the fundamental spirit of the clinical trial management standards and Helsinki Declaration (revised 2013). In addition, this study complies with ICH-GCP and KGCP and will start research after IRB approval. If violation of human rights occurs during this study, it will be notified to the Clinical Trial Review Committee/ Institutional Review Board. Information that can identify the research subject will be confidential by the researcher, and research data will be recorded with initials and coded research subject identification information. In addition, research subjects and research information will be stored on a computer with limited access, and research will be explained and informed consent will be obtained in an independent space to protect the personalities of the study subjects. Even if the results of the clinical trial are published, the personal information of the study subjects will remain confidential.

1. **Subject's consent process**

- Researcher Who will explain and obtain consent from the study subjects: Research Manager, Research Officer
- Person who provides consent: Subject or representative
- Time for the explanation of the research process and consent acquisition: 30 minutes (acquired when visiting the laboratory/medical center)
- Method to minimize the possibility of forced or unjustified impact: Explain that participation in the study is only possible by the patient's own voluntary will, and that even if patient doesn’t want to participant, it will have no effect on future treatment processes.
- Language used by researchers in the process of explaining research and obtaining consent: Korean
- Language that the subject or representative can understand: Korean

1. **Compensation plan for study subjects**

Not applicable

1. **Personal information protection plan for study subjects**

The information of all subjects participating in this study will be managed in accordance with the relevant laws and regulations. It will be kept strictly confidential so as not to be exposed to third parties except related researchers, IRBs and research funding agencies, and will conduct research by coding or anonymizing so as not to be connected to the patient's personal information. In this study, the patient's address or contact information won’t be collected or managed separately, and the data file will not display the patient's personal identification number or patient's hospital ID. All data files must have a separate password to be managed by locking for security.

According to Article 15 of the Enforcement Rules of the Bioethics Act, research-related records shall be kept for three years from the end of the study, and documents or data files that have passed the storage period shall be destroyed in accordance with Article 16 of the Enforcement Decree of the Personal Information Protection Act. If storage is required for more than three years for follow-up research, records, or accumulation, a separate request will be made to the IRB to obtain permission to extend the storage period of data before the expiration of the validity period.

1. **Additional protective methods for vulnerable research subjects**

Not applicable

1. **Management, storage, and disposal plan for collecting human-derived materials, genetic information, etc.**

Not applicable

1. **Reference**

1. Benjamin EJ WP, D'Agostino RB, Silbershatz H, Kannel WB, Levy D. Impact of atrial fibrillation on the risk of death: the Framingham Heart Study. Circulation. 1998;98(10):946-52.

2. January CT, Wann LS, Alpert JS, Calkins H, Cigarroa JE, Cleveland JC, Jr., et al. 2014 AHA/ACC/HRS guideline for the management of patients with atrial fibrillation: a report of the American College of Cardiology/American Heart Association Task Force on practice guidelines and the Heart Rhythm Society. Circulation. 2014;130(23):e199-267.

3. Colilla S, Crow A, Petkun W, Singer DE, Simon T, Liu X. Estimates of current and future incidence and prevalence of atrial fibrillation in the U.S. adult population. Am J Cardiol. 2013;112(8):1142-7.

4. Kim D, Yang PS, Jang E, Yu HT, Kim TH, Uhm JS, et al. 10-year nationwide trends of the incidence, prevalence, and adverse outcomes of non-valvular atrial fibrillation nationwide health insurance data covering the entire Korean population. Am Heart J. 2018;202:20-6.

5. Kirchhof P, Camm AJ, Goette A, Brandes A, Eckardt L, Elvan A, et al. Early Rhythm-Control Therapy in Patients with Atrial Fibrillation. N Engl J Med. 2020;383(14):1305-16.

6. Kareti KR, Chiong JR, Hsu SS, Miller AB. Congestive heart failure and atrial fibrillation: rhythm versus rate control. J Card Fail. 2005;11(3):164-72.

7. Heist EK, Ruskin JN. Atrial fibrillation and congestive heart failure: risk factors, mechanisms, and treatment. Prog Cardiovasc Dis. 2006;48(4):256-69.

8. Anter E, Jessup M, Callans DJ. Atrial fibrillation and heart failure: treatment considerations for a dual epidemic. Circulation. 2009;119(18):2516-25.

9. SOLTI F, VECSEY T, KÉKESI V, JUHÁSZ-NAGY A. The effect of atrial dilatation on the genesis of atrial arrhythmias. Cardiovascular Research. 1989;23(10):882-6.

10. Frank Bode AK, Raymond L. Woosley, and Michael R. Franz. Gadolinium Decreases Stretch-Induced Vulnerability to Atrial Fibrillation. Circulation. 2000;101(18):2200-5.

11. Dirk J. Beuckelmann MMN, MD; and Erland Erdmann, MD. Intracellular Calcium Handling in Isolated Ventricular Myocytes From Patients With Terminal Heart Failure. Circulation. 1992;85(3):1046-55.

12. Ohkusa T, Ueyama T, Yamada J, Yano M, Fujumura Y, Esato K, et al. Alterations in cardiac sarcoplasmic reticulum Ca2+regulatory proteins in the atrial tissue of patients with chronic atrial fibrillation. Journal of the American College of Cardiology. 1999;34(1):255-63.

13. McMurray JJV, Solomon SD, Inzucchi SE, Kober L, Kosiborod MN, Martinez FA, et al. Dapagliflozin in Patients with Heart Failure and Reduced Ejection Fraction. N Engl J Med. 2019;381(21):1995-2008.

14. Packer M, Anker SD, Butler J, Filippatos G, Pocock SJ, Carson P, et al. Cardiovascular and Renal Outcomes with Empagliflozin in Heart Failure. N Engl J Med. 2020;383(15):1413-24.

15. Faul F, Erdfelder E, Buchner A, Lang A-G. Statistical power analyses using G* Power 3.1: Tests for correlation and regression analyses. Behavior research methods. 2009;41(4):1149-60.

16. Andrade JG, Deyell MW, Verma A, Macle L, Champagne J, Leong-Sit P, et al. Association of atrial fibrillation episode duration with arrhythmia recurrence following ablation: a secondary analysis of a randomized clinical trial. JAMA network open. 2020;3(7):e208748-e.

17. Fernandes GC, Fernandes A, Cardoso R, Penalver J, Knijnik L, Mitrani RD, et al. Association of SGLT2 inhibitors with arrhythmias and sudden cardiac death in patients with type 2 diabetes or heart failure: A meta-analysis of 34 randomized controlled trials. Heart rhythm. 2021.

18. Andrade JG, Wells GA, Deyell MW, Bennett M, Essebag V, Champagne J, et al. Cryoablation or drug therapy for initial treatment of atrial fibrillation. New England Journal of Medicine. 2021;384(4):305-15.
