# Supplementary material for: Protocol of BEYOND trial: Clinical BEnefit of sodium-glucose cotransporter-2 (SGLT-2) inhibitors in rhYthm cONtrol of atrial fibrillation in patients with diabetes mellitus
Source: PLoS One. 2023 Jan 18;18(1):e0280359. doi: 10.1371/journal.pone.0280359 (PMC9847966; doi:10.1371/journal.pone.0280359)
Supplement: S4 File — (PDF) [file pone.0280359.s004.pdf]

통지서

※ 본 과제의 문서보존기간은 3 년입니다.						
수신	의뢰(지원)기관	내부과제				
	연구책임자	순환기내과 박준범				
IRB File No.		EUMC 2021-08-034-002	심사내용	시정계획서	통지일자	2021.10.05
연구과제명	국문	심방세동과 동반된 당뇨 환자에서의 SGLT-2 inhibitor 사용이 미치는 효과				
	영문	The effect of SGLT-2 inhibitor in patient with atrial fibrillation and diabetes mellitus				
임상시험코드			Study Nick Name			

연구분류1	☐ 약물 ☐ 생물학적 제재 ☐ 세포치료제 ☐ 건강기능식품
	☐ 의료시술 ☐ 의료기기 (○ 1등급 ○ 2등급 ○ 3등급 ○ 4등급)
	■ 해당사항없음
연구분류2	■ 인간대상연구 ☐ 인체유래물(검체)연구 ☐ 의무기록연구
	☐ 유전자연구 ☐ 유전자치료
	☐ 배아연구 ☐ 체세포복제배아연구 ☐ 줄기세포주연구
	☐ 기타 ( )
연구분류3	◉ 전향적 연구 ○ 후향적 연구 ○ 전향적 & 후향적 병행연구
연구분류 4	■ 중재연구 ☐ 설문조사 ☐ 자료분석 및 분석연구
	☐ 관찰연구 (☐ 단면조사연구 ☐ 환자대조군연구 ☐ 코호트 연구)
	☐ 기타 ( )
연구분류 5	☐ 인간을 대상으로 하지 않는 연구 Non-clinical study (in vitro. in vivo preclinical study)

일반명			상품명			
전체피험자증례수	전체	720 명	국내	720 명	본원	400 명
연구승인기간	2021.10.05 ~ 2022.10.04					
지원의뢰기관	기관명	내부과제	대표(직위)		성명	
제출서류목록						

<table>
<tr><td>제출서류목록</td><td colspan="3">(첨부) 연구계획서 요약 [] []
(첨부) 연구계획서(protocol) [Ver1.0] []
(첨부) 대상자 설명문 및 동의서 [Ver1.0] []
(첨부) 연구윤리교육이수증 [] []
(첨부) 연구자 최근 이력 또는 기타경력에 관한 문서 [] []
(첨부) 변경대비표 [] []
(첨부) 별첨1. 당뇨 약물 정보 [] []</td></tr>
<tr><td>관련근거</td><td>평가일자</td><td colspan="2">2021.10.05</td></tr>
<tr><td>중간보고시기</td><td>2022년 08월 04일</td><td>비고</td><td>* 지속심의 빈도 : 12개월</td></tr>
<tr><td>심사결과</td><td colspan="3">◉ 승인 ○ 시정승인</td></tr>
<tr><td>심사결과</td><td colspan="3">제출한 시정계획을 확인하여, 연구의 수행을 1년간 '승인'합니다.

〈시정답변〉
1. 연구계획서 Ver1.0(version변경없음)
1) 연구배경의 심방세동 유병율을 현재 시점을 기준으로 서술 변경.
2) 5page, '4) 목표한 대상자 수 및 산출근거'에 대상자 수 산출근거 추가함.
- 전체 목표대상자 수 변경
: 총 720명(투여 360명, 대조 360명) → 총 704명(투여 352명, 대조 352명)
3) 12개월째 최종 추적 관찰시 Left atrial size, NT-pro BNP, quality of life (AFEQT)를 조사하도록 연구방법 일치시킴
4) 9page, '연구도식도'의 선정 / 제외 기준을 계획서의 내용과 동일하도록 수정함.
5) 대상자 당뇨관리 계획에 대한 항목 추가 : 10page, '13) 연구대상자의 당뇨 관리 계획'
6) 연구에서 사용되는 경구혈당강하제의 약물 정보를 '별첨1. 당뇨약물 정보 Ver1.0'으로 추가함.

2. 연구대상자 설명문 및 동의서 Ver1.0
1) 대상자 이해를 돕기위해 영어 표현에 한글 병기를 추가하거나 한글 용어로 수정함.
2) 목표례 변경사항 반영
3) '12. 당뇨관리계획' 항목 신설 : 대상자 당뇨관리에 대한 설명 추가함.

3. 연구계획서 요약
1) 계획서 변경사항에 따라 연구 목표, 대상자 수 산출 근거 , 연구방법 등 수정함.

〈추가 변경사항〉
1. 연구계획서 Ver1.0
1) 공동연구자 추가 : 내과 전보경
2) 연구목적을 구체화하여 기술함.
- 1차 목표를 '항부정맥제 사용 1년 또는 전극도자 절제술 후 1년까지의</td></tr>
</table>

심사결과	′심방세동 재발률 (AF recurrence)′을 평가하는 것으로 함.<br>3) 당뇨병 관련 당화혈색소의 세부 선정기준 추가함.<br>- 경구 약제를 복용하지 않고 있는 경우 HbA1c 7.5% 이상<br>- 경구 혈당강하제(metformin 단독 또는 2제 / 3제 요법)를 3개월 이상 사용한 경우 HbA1c 7.0% 이상<br>4) 8PAGE, 안전성 평가방법 구체화함.<br><br>2. 연구대상자 설명문 및 동의서 Ver1.0(version 변경없음)<br>1) 공동연구자 정보 추가함.<br>[안내]<br>1. 본 연구의 IRB 승인기간은 2022년 10월 04일까지이며, 승인기간 이후에도 연구를 지속하기 위해서는 e-IRB 게시판 > 양식함 > ′[서식 11]지속심의(중간보고)의뢰서′를 제출하여 연구의 지속적 수행을 승인 받으시기 바랍니다.<br>(IRB 승인기간 만료일 전 과제가 종료되었을 경우, ′[서식 12]종료보고의뢰서′를 제출하여 과제 종료하여 주십시오.)<br>2. 중간보고 제출은 2022년 08월 04일부터 가능합니다.

1. 본 기관생명윤리위원회는 생명윤리 및 안전에 관한 법, 의약품 등의 안전에 관한 규칙, 의료기기법 시행규칙, 헬싱키 선언 및 국제임상시험통일안(ICH-GCP) 등 관련 법규를 준수합니다.
2. IRB 승인 이전에 연구대상자의 연구참여는 금지되며, IRB 승인 받은 동의서를 사용해야 하고 승인받은 모집공고문을 사용해야 합니다.
3. IRB의 승인은 1년을 초과할 수 없으며, 1년 이상 연구를 지속하고자 하는 경우에는 본 위원회에서 지정한 중간보고시기에 중간보고를 해야 하며, 연구 종료 시에는 종료보고서를 제출해야 합니다.
4. 연구대상자에게 발생한 즉각적 위험 요소의 제거가 필요하여 원 계획서와 다르게 연구를 실시해야 하는 경우, 연구대상자에게 발생하는 위험요소를 증가시키거나 연구의 실시에 중대한 영향을 미칠 수 있는 변경사항, 예상하지 못한 중대한 이상약물반응에 관한 사항, 연구대상자의 안전성이나 임상시험 실시에 부정적인 영향을 미칠 수 있는 새로운 정보에 관한 사항은 IRB에 신속히 보고해야 합니다.
5. 본 통지서는 KGCP 제13조 제①항에 따른 심사통보서로 사용할 수 있으며, 기관생명윤리위원회에 기록된 내용과 동일 합니다.
6. 심의결과 '승인'이 아닌 경우에는 답변서를 제출해야 하며(심의일 기준) 6개월 이내에 이루어져야 하며 '보완'은 정규심의로 진행됩니다(초기심의 시 심의된 패널과 동일한 패널로 접수됨).
7. 직인(서명)이 기재되지 않은 통지서는 무효입니다.
(위원회가 연구계획서를 승인하지 않은 경우 즉, 반려, 중지 / 보류의 결정을 통보 받은 경우 4주 이내에 이의신청사유와 함께 필요한 서류를 위원회에 제출하여 이의신청을 할 수 있습니다. 단, 동일연구에 대한 이의신청은 2번까지 가능)
8. 승인 받은 연구에 대하여 기관의 내부점검 및 외부의 실태조사를 받을 수 있습니다.
9. 헬싱키선언(제19조)에 따라 모든 임상시험은 첫 피험자 모집하기 전 공개적으로 접근이 가능한

임상연구등록시스템(primary registry)에 등록하여 이를 공개하여야 하며, 예를 들어, 질병관리본부에서 운영하는 임상연구정보서비스(CRIS, https: / / cris.nih.go.kr)를 이용하실 수 있습니다.

10. 본 과제의 연구자와 이해상충(COI, Conflict of interest)이 있는 위원은 심의결정과정에 참여하지 않습니다.

이대목동병원 IRB
