# Supplementary material for: Protocol of BEYOND trial: Clinical BEnefit of sodium-glucose cotransporter-2 (SGLT-2) inhibitors in rhYthm cONtrol of atrial fibrillation in patients with diabetes mellitus
Source: PLoS One. 2023 Jan 18;18(1):e0280359. doi: 10.1371/journal.pone.0280359 (PMC9847966; doi:10.1371/journal.pone.0280359)
Supplement: S5 File — (PDF) [file pone.0280359.s005.pdf]

Approval Latter

* The archiving period for this project is three years.						
Receiving	Commissioned organization	Internal task				
	Research manager	Junbeom Park, Department of Cardiology				
IRB File No,		EUMC 2021-08-034-002	Contents of review	Corrected plan	Approval Date	2021.10.05
Title	Kor	심방세동과 동반된 당뇨 환자에서 SGLT-2 사용이 미치는 효과				
	Eng	The effect of SGLT-2 inhibitor in patient with atrial fibrillation and diabetes mellit us				
Clinical trial code			Study Nick Name			

Research classification 1	□ Drug □ Biological agents □ Cell therapy □ Dietary supplement					
	□ Medical procedure □ Medical device (○ first ○ second ○ third ○ fourth grade)					
	■ Not applicable					
Research classification 2	■ Human subject study □ Human-derived material study □ Medical record study					
	□ Gene study □ Gene treatment					
	□ Embryo study □ somatic cloning embryo study □ Stem cell study					
	□ Etc. ( )					
Research classification 3	● Prospective ○ Retrospective ○ Combined					
Research classification 4	■ Interventional study □ Survey □ Data analysis and analysis research					
	□ Observational study (□ cross-sectional study □ case-control study □ cohort study)					
	□ Etc. ( )					
Research classification 5	□ Non-clinical study (in vitro, in vivo preclinical study)					
General name	–		Product name			
Number of subjects	Total	720	Domestic	720	This hospital	400
Approval period	2021. 10. 05 ~ 2022. 10. 04					
Cmmissioned organization	Name of organization		Representative (Position)		Name of rresentative	
List of attached documents						

<table>
<tr><td>List of attached documents</td><td colspan="4">(attached) Summary of research plan
(attached) Research plan (protocol) [Ver. 1.0]
(attached) Explanation for subjects and informed consent [Ver. 1.0]
(attached) Certificate of completion of research ethics education
(attached) Articles of the researcher's recent history or other background
(attached) Change comparison table
(attached) additional attachment 1: information of diabetes medication</td></tr>
<tr><td>Relevant basis</td><td>Evaluation date</td><td colspan="3">2021. 10. 05</td></tr>
<tr><td>Interim report date</td><td colspan="2">2022. 08. 04</td><td>NOTE</td><td>* frequency of review : 12 month</td></tr>
<tr><td>Result</td><td colspan="4">● Approve ○ Approval after correction</td></tr>
<tr><td>Result</td><td colspan="4">We checked the submitted corrective plan and "approve" this research for one year.

<Corrective reply>

1. Research plan [Ver. 1.0] (No version change)
1) The description of the prevalence of atrial fibrillation in the background was changed to current time.
2) Basis for calculating the number of subjects was added at page 5, 4) Target number of subjects and basis for calculation
- Changing the total number of targets
: total 720 people (360 people as SGLT-2 inhibitor administration group, 360 people as control group) -> total 704 people (352 people as SGLT-2 inhibitor administration group, 352 people as control group)
3) The study methods were equalized to investigate left atrial size, NT-pro BNP and quality of life (AFEQT) at final follow-up for 12 months.
4) The inclusion/exclusion criteria at schematic diagram (9 page) revised to the same as the contents of the plan.
5) Items to the subject's diabetes management plan were added; page 11, 13) Study subjects' diabetes management plan
6) Additional drug information of oral hypoglycemic agents used in the study was added; additional attachment 1: information of diabetes medication

2. Explanation for subjects and informed consent [Ver. 1.0] (No version change)
1) In order to help the subject understand, Korean is used in combination with English expressions or modified into Korean terms.
2) Changes in target number of subjects was reflected.
3) Category of '12. diabetes management plan' was created; add an explanation of the subject's diabetes management plan

3. Summary of research plan
1) According to changes in the plan, research goals, basis for calculating the number of subjects and research methods are revised.</td></tr>
</table>

Result	<Additional modifications><br><br>1. Research plan [Ver. 1.0] (No version change)<br>1) Added a co-researcher: Bo Kyung Jeon, Department of internal medicine<br>2) Materialized research objectives<br>- Primary goal was changed to evaluating recurrence rate of atrial fibrillation (AF recurrence) up to one year after using antiarrhythmic drugs of taking ablation.<br>3) Detailed selection criteria for glycated hemoglobin related to diabetes were added.<br>- HbA1c ≥ 7.5%, if patient have no oral DM medication<br>- HbA1c ≥ 7.0%, if patient already have oral hypoglycemic agents (metformin alone or double/triple-agent therapy) more than 3 months<br>4) Made more precisely; page 8, safety evaluation methods<br><br>2. Explanation for subjects and informed consent [Ver. 1.0] (No version change)<br>1) Added information of co-researcher.<br><br>[Notice]<br>1. The IRB approval period for this study is until October 4, 2022, and in order to continue the study after the approval period, please submit an 'eIRB board > form storage box > [Form 11] Interim Report Request' and obtain approval for the continuous study.<br>2. Submission of interim report will be available from August 4, 2022.

1. This IRB complies with relevant laws and regulations such as the Bioethics and Safety Act, the Enforcement Rules of the Medical Device Act, the Helsinki Declaration, and the International Clinical Trial Uniformity (ICH-GCP).

2. Prior to IRB approval, the participation of research subjects is prohibited, and consent forms and recruitment notices approved by IRB must be used.

3. IRB approval cannot exceed one year, and if the study is to be continued for more than one year, an interim report must be made at the time specified by this committee, and a final report must be submitted at the end of the study.

4. If the study should be conducted differently from the original plan to eliminate immediate risks arising from the study, the things below that should be reported to IRB.; Changes that may increase the risk factors to participants or have a significant impact on the conduct of the study, major adverse drug reactions which are not expected, and new information that may negatively affect the study.

5. This notification can be used as an notification of examination pursuant to Article 13 (1) of the KGPC and is the same as what is recorded in the Institutional Review Board.

6. Letter of reply must be submitted if it is not 'approved', and the submission must be made within six months (as of the deliberation date). 'Supplementation' will be carried out by regular deliberation. (Review panel will be consisted as same as initial)

7. Notification without a seal (signature) is invalid.

(If the committee does not approve the research plan, that is, if it is decided to reject or suspend, it can file an objection with the relevant documents to the committee within four weeks.)

[redacted]

8. You can receive an internal inspection and an external survey of approved studies.

9. According to the Helsinki Declaration (Article 19), all clinical trials must be registered and disclosed in a publicly accessible clinical research registration system before recruiting the first subject. For example, you can use the clinical research information service operated by the Korea Centers for Disease Control (CRIS, https://cris.nih.or.kr).

10. Members who have conflicts of interest (COI) with the researcher of this project do not participate in the deliberation decision process.

이대목동병원 IRB

Institutional review board of Ewha Womans University Mokdong Hospital
