# Supplementary material for: Protocol of BEYOND trial: Clinical BEnefit of sodium-glucose cotransporter-2 (SGLT-2) inhibitors in rhYthm cONtrol of atrial fibrillation in patients with diabetes mellitus
Source: PLoS One. 2023 Jan 18;18(1):e0280359. doi: 10.1371/journal.pone.0280359 (PMC9847966; doi:10.1371/journal.pone.0280359)
Supplement: Attachment — Submitted filename: Respond to Reviewers.docx [file pone.0280359.s006.docx]

**Respond to Journal Requirements**

1. Please ensure that your manuscript meets PLOS ONE's style requirements, including those for file naming. The PLOS ONE style templates can be found at

https://journals.plos.org/plosone/s/file?id=wjVg/PLOSOne_formatting_sample_main_body.pdf and

https://journals.plos.org/plosone/s/file?id=ba62/PLOSOne_formatting_sample_title_authors_affiliations.pdf

- We have re-checked and edited our manuscript set, including the naming of files to meet the PLOS ONE's style requirements.

2. Thank you for stating the following in the Acknowledgments Section of your manuscript:

“This trial is supported by Basic Science Research Program through the National Research Foundation of Korea (NRF) funded by the Ministry of Science, ICT & Future Planning (NRF-2017R1E1A1A01078382), and by the Korea Medical Device Development Fund grant funded by the Republic of Korea government (the Ministry of Science and ICT, the Ministry of Trade, Industry and Energy, the Ministry of Health & Welfare, the Ministry of Food and Drug Safety) (Project Number: 9991006899).”

We note that you have provided funding information that is currently declared in your Funding Statement. However, funding information should not appear in the Acknowledgments section or other areas of your manuscript. We will only publish funding information present in the Funding Statement section of the online submission form.

Please remove any funding-related text from the manuscript and let us know how you would like to update your Funding Statement. Currently, your Funding Statement reads as follows:

“This trial is supported by Basic Science Research Program through the National Research Foundation of Korea (NRF) funded by the Ministry of Science, ICT & Future Planning (NRF-2017R1E1A1A01078382), and by the Korea Medical Device Development Fund grant funded by the Republic of Korea government (the Ministry of Science and ICT, the Ministry of Trade, Industry and Energy, the Ministry of Health & Welfare, the Ministry of Food and Drug Safety) (Project Number: 9991006899).”

Please include your amended statements within your cover letter; we will change the online submission form on your behalf.

- We deleted all the funding-related text in the manuscript – in the Materials and Methods and Acknowledgements – as requested. We would like to retain the original Funding Statement.

3. Please include your full ethics statement in the ‘Methods’ section of your manuscript file. In your statement, please include the full name of the IRB or ethics committee who approved or waived your study, as well as whether or not you obtained informed written or verbal consent. If consent was waived for your study, please include this information in your statement as well.

- We re-organized and reinforced the ethics statement that you requested in the ‘Study status and organization’ subsection of the Methods section. The exact name of the ethics committee who approved our study design – the Institutional Review Board of Ewha Womans University Mokdong Hospital – has been indicated. A statement regarding receiving informed written consent from patients has also been included.

4. We note that the original protocol that you have uploaded as a Supporting Information file contains an institutional logo. As this logo is likely copyrighted, we ask that you please remove it from this file and upload an updated version upon resubmission.

- We have deleted the institutional logo in supplement files 2 and 3 and re-uploaded the modified files.

**Respond to Reviewers' comments**

Reviewer #1: The authors have clearly mentioned the details of ongoing trial protocols taking into account every aspect of the study and I have no major comments to make.

Just a minor comment:

1. The introduction section can be improvised a bit with a better flow of writing in the light of some recent findings albeit indirect but important.

"There is emerging evidence that suggests SGLT2I use may be associated with lesser episodes of AF compared to non-users. Example - incidental lesser AF amongst SGLT2I users as from USFDA AERS (Bonora et al. SGLT-2 inhibitors and atrial fibrillation in the Food and Drug Administration adverse event reporting system. Cardiovasc Diabetol 2021. https://doi.org/10.1186/s12933-021-01243-4) and, a pooled meta-analysis of 8 CV/Renal outcome trials (Okunrintemi et al. Sodium-glucose co-transporter-2 inhibitors and atrial fibrillation in the cardiovascular and renal outcome trials. Diabetes Obes Metab. 2021;23:276-280). These pieces of evidence suggest that an RCT is necessary to come to such a conclusion and a direct trial such as the BEYOND trial would help to this end.

- We have briefly mentioned the merits of our prospective study on the relationship between SGLT-2 inhibitors and AF in the Discussion section. However, there was a lack of references to recent studies; we therefore revised and added reports of several recent findings in the Introduction and Discussion sections according to your much-appreciated advice.

Reviewer #2: The manuscript is inspiring and well-written, elaborating a clinical trial rationale in which authors tried to test out if SGLT-2 inhibitors might be able to improve the clinical outcome of AF with DM. However, there are some questions confusing me:

1. As mentioned in the context, AF and CHF are mutual risk factors, how to distinguish between AF patients and CHF patients (or patients with multiple risk factors for CHF)? I mean, CHF patients will more likely to benefit from SGLT-2I as shown in EMPEROR-Reduced and EMPEOR-Preserved, so, naturally, patients with CHF and AF are more likely to benefit from the SGLT-2 inhibitor, however, I did not see NT-proBNP or CHF in the exclusion criteria, is it reasonable to propose that a substantial number of patients in your inclusion process are complicated with CHF or multiple risk factors of CHF?

- We did not include NT-proBNP or CHF separately, according to the exclusion criteria. Therefore, it is believed that registered subjects will be mixed with a certain percentage of groups with and without HF. We anticipated that this population will be able to produce results that better reflect the real world, and as you mentioned, HF, a strong confounding factor, can be considered in future analyses, such as multivariable Cox regression analysis or subgroup analysis.

2. I am just wondering about whether AF evaluation will be presented in your following articles, such as AF type, symptom severity, severity of AF burden, stroke risk, etc. Since they are important baseline characteristics and should be balanced in the intervention and control groups.

- The secondary outcomes of our study included AF burden, sinus rhythm maintenance, and EHRA symptom score. Therefore, the subjects participating in our study will be checked for 12-lead ECG, 24-h Holter ECG, and EHRA symptom scores at the beginning and end of follow-up. Through these clinical data, baseline data and changes in subjects' AF characteristics can be presented at the result-reporting stage.
